# Synthesis, Characterizations, Anti-Diabetic and Molecular Modeling Approaches of Hybrid Indole-Oxadiazole Linked Thiazolidinone Derivatives

**DOI:** 10.3390/ph17111428

**Published:** 2024-10-24

**Authors:** Shoaib Khan, Tayyiaba Iqbal, Rafaqat Hussain, Yousaf Khan, Zanib Fiaz, Fazal Rahim, Hany W. Darwish

**Affiliations:** 1 Department of Chemistry, Abbottabad University of Science and Technology (AUST), Abbottabad 22500, Pakistan; tayyiaba.iqbal23@gmail.com (T.I.); cuttlefishzee@gmail.com (Z.F.); 2 College of Biology, Hunan University, Changsha 410082, China; rafaqathussain0347@gmail.com; 3 Department of Chemistry, COMSATS University Islamabad, Islamabad 45550, Pakistan; yousaf7n@gmail.com; 4 Department of Chemistry, Hazara University, Mansehra 21300, Pakistan; fazalrahim@gmail.com; 5 Department of Pharmaceutical Chemistry, College of Pharmacy, King Saud University, P.O. Box 2457, Riyadh 11451, Saudi Arabia; hdarwish@ksu.edu.sa

**Keywords:** oxadiazole, thiazolidinone, diabetes miletus, molecular docking, ADMET

## Abstract

**Objective:** To synthesize hybrid compounds of indole and oxadiazole in search of highly effective anti-diabetic therapeutic agent. **Methods:** With the goal of advancing diabetes research, our group designed and synthesized a library of 15 compounds based on indole-derived oxadiazole bearing varied substituted thiazolidinone via a multistep synthetic route. ^13^C-NMR, ^1^H-NMR and HREI-MS were applied for the characterization of all the synthesized compounds. Their biological inhibitory activity against diabetic enzymes, i.e., α-amylase and α-glucosidase was also determined. **Results:** Compound **7, 9 and 15 exhibited** excellent inhibition against α-amylase and α-glucosidase than the standard acarbose (IC_50_ = 8.50 ± 0.10 µM for α-amylase and 9.30 ± 0.30 µM for α-glucosidase. To ensure the inhibitory actions of these potent analogs in molecular docking, an in silico approach was used. To determine the drug likeness of the reported analogs, an ADMET investigation was also carried out to explore the nature of the designed compounds if used as a drug. **Conclusion:** Fluoro-substituted analog 15 has stronger inhibition profile against both enzymes. All the potent compounds can be used as effective anti-diabetic therapeutic agents in future.

## 1. Introduction

Diabetes mellitus (DM) is a chronic disorder involving an increased level of blood glucose, often termed hyperglycemia. Millions of people worldwide are affected by this disease [1,2,3]. In diabetes the body is not able to utilize insulin or, in some cases, is not able to produce insulin. Due to the imbalanced blood glucose levels, the patient suffers from severe health complications that can even lead to death. This silent killer significantly affects patients’ quality of life, making it an important issue to be addressed. Sudden stroke, complications like retinopathy, blindness, kidney issues like kidney failure, problems with metabolism, and issues in the blood circulatory systems eventually leading to death are major side effects linked with diabetes [4,5]. The onset of this condition is due to the unstable activity of the enzyme α-amylase, which is known to degrade complex carbohydrates into small molecules that are then converted into absorbable sugar molecules through the actions of another enzyme, α-glucosidase [6]. To control the actions of these enzymes, drugs like Acarbose and Miglitols are available on the market [7]. These drugs are used to inhibit the actions of the mentioned enzymes, which can ultimately control the concentration of sugar in the blood, controlling the diabetes. However, their side effects are a major concern [8,9,10]. This need to produce more medications for diabetes has attracted the attention of scientists worldwide [11]. The effectiveness of the drug and its cost, along with its side effects, are major considerations in this search.

For treating such a fatal disease, moieties incorporating heteroatoms were found to effectively inhibit the actions of uncontrolled enzymatic activity. In the field of drug design, a heterocyclic moiety, termed an indole, was found to be very effective in various studies conducted by researchers [12,13,14,15,16]. Indole and its derivatives are also reported to be anti-malarial [17] and anti-phlogistic agents [18,19]. They were also found to be very active against bacteria [20] and exhibited excellent anti-diabetic potential [21]. They also have great effectiveness against allergies [22] and have emerged as good cytotoxic drugs [23,24]. Moreover, these compounds’ anti-HIV activity [25] and ability to regulate blood pressure have also been reported [26]. To obtain active indole-based derivatives, a substitution is attached at carbon-3 [27,28,29]. The substituted indole derivatives also possess anti-hypoglycemic and anti-inflammatory properties [30,31,32].

**Figure 1:**
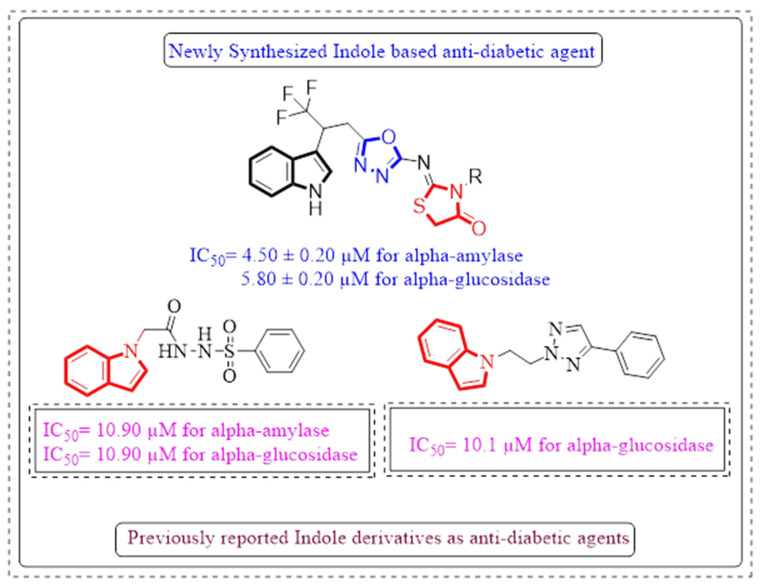
Rationale study [33,34] expressing the potential of the novel compound as an anti-diabetic agent.

Keeping the biological importance of indole and its derivatives in view, in this work, our group designed indole-based derivatives for use as antidiabetic inhibitors. The inhibitory potential of the novel compounds was also compared to previously reported compounds in the literature. Interestingly, the previously reported indole-based anti-diabetic agents were found to have lower inhibitory potential. This analysis suggests the biological potency of these novel compounds and presents an effective therapeutic agent for diabetes mellitus. The rationale of the current work is presented in Figure 1.

## 2. Results and Discussion

### 2.1. Chemistry

**Scheme 1:**
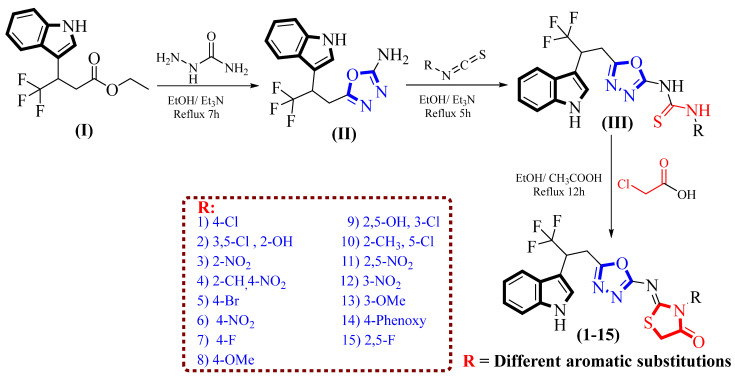
Designed route to obtain hybrid indole–oxadiazole linked thiazolidinone derivatives.

To synthesize hybrid indole–oxadiazole-linked thiazolidinone derivatives, an indole derivative (I) and semicarbazide were reacted in ethanol under reflux for 7 h, which afforded the first intermediate [indole-derived oxadiazole-bearing amine] (II). This newly collected intermediate (II) was further reacted with varied substituted iso-thiocyanate. This was the second step of the aforementioned synthetic route, using ethanol as a solvent. Triethylamine (ET3N) was also added as a catalyst in the reaction mixture. After 5 h reflux, a second intermediate [indole-derived oxadiazole-bearing thiourea derivative] (III) was afforded. This second intermediate (III) was then treated with 2-choroacetic acid to obtain the cyclized final product: hybrid indole–oxadiazole-linked thiazolidinone derivatives (**1**–**15**). The final products were synthesized under reflux for 12 h in ethanol. The synthetic procedure of novel efficient route is given in Scheme 1. These products were then collected at low pressure by evaporating the ethanol, and fine powder products were collected. In order to remove impurities, all the synthesized novel compounds were washed using n-hexane and dried at reduced pressure. The compounds were synthesized in a good yield ranging between 63 and 81%, as shown in Table 1. The collected samples were structurally investigated by 13C-NMR and 1H-NMR, and their mass was calculated via HREI-MS. These compounds were then biologically assessed for their drug potential, which was also validated by conducting an in silico study.

### 2.2. Structure-Activity Relationship (SAR)

In order to obtain biological profiles, all the derivatives were screened against enzymes α-amylase along and α-glucosidase. These derivatives were investigated against both enzymes, and found to exhibit distinct IC_50_ values (Table 2). Compounds having reactive substitution and the presence of a lone pair were found to have an excellent biological profile.

Compounds having Cl-atom as attachment show quite a good profile against both enzymes. Derivative-**1**, having *p*-chloro, demonstrated a noteworthy level of activity, with an inhibition concentration of 13.10 ± 0.40 µM against the alpha-amylase enzyme and 12.30 ± 0.10 µM against alpha-glucosidase. We know that halogens are ring activators, shifting the electronic cloud toward the phenyl ring, and this contributes to the active nature of derivative-**1**. The second derivative, which has *o*-hydroxyl and *m*-chloro as represented in derivative-**2**, showed an inhibition concentration of 8.20 ± 0.10 µM against alpha-amylase and 9.10 ± 0.30 µM against alpha-glucosidase. This compound was found to be more active than compound-**1**, which might be due to the presence of hydroxyl and chlorine groups, which also activate the ring developing several types of interactions with the targeted enzymes. The hydroxyl group also has the potential for hydrogen bond formation, which results in a good inhibition profile for the compound.

Compound-**9**, having two hydroxyl groups at the *meta* and *ortho* positions and a chloro group at the other *meta* position, was found to be active against alpha-amylase, with an IC_50_ value of 7.30 ± 0.20 µM, as well as against alpha-glucosidase, with an inhibition value of 7.60 ± 0.20 µM. Both hydroxyl groups can generate hydrogen bond interaction by using a hydrogen atom and a lone pair of oxygen. A chlorine atom can also activate the ring along with the hydroxyl group, making compound-**9** a good inhibitor against both enzymes.

**Figure 2:**
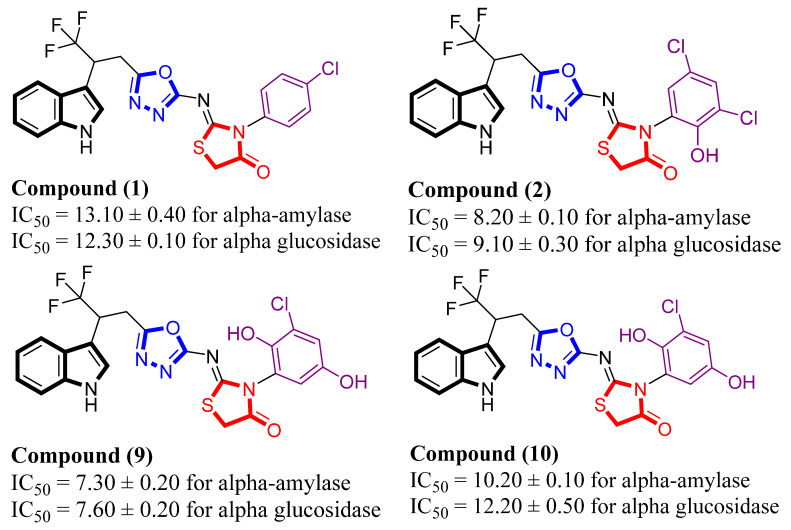
Inhibitory activity of derivatives **1**, **2**, **9** and **10**.

Compound-**10**, having an *m*-chloro and *o*-methyl substitution, showed some activity, with IC_50_ values of 10.20 ± 0.10 µM and 12.20 ± 0.50 µM against targeted enzymes. The decline in the activity of this compound might be due to the presence of the bulky methyl group, which has a hydrophobic character and causes steric hindrance and resists the ligand–protein interaction. Inhibitory profile of compound-**1**, **2**, **9** and **10** is given in Figure 2.

Derivative-**3**, having nitro substitution at its *ortho* position, had an inhibition concentration of 15.30 ± 0.20 µM against alpha-amylase and 14.50 ± 0.20 µM against alpha-glucosidase. This might be due to the electron-withdrawing nature of NO_2_, shifting the electronic cloud towards itself and deactivating the ring for further interaction. The position of the nitro group also reduces interactivity under the NOE effect and thus resists the nitro moiety to attract the amino acid of the targeted enzyme, resulting in the lower inhibition rate of this compound.

**Figure 3:**
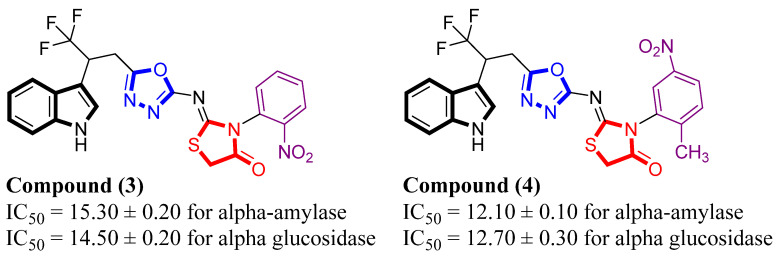
Inhibitory activity of derivatives-**3** and **4**.

Compound-**4**, having a nitro group at the *meta* position and a methyl substitution at the *ortho* position, has an inhibition rate of 12.10 ± 0.10 µM against alpha-amylase and 12.70 ± 0.30 µM against glucosidase. As the methyl group is electron-donating in nature, it accelerates the activity of the ring and develops a number of interactions. Inhibitory activity of derivatives-**3** and **4** in given in Figure 3.

**Figure 4:**
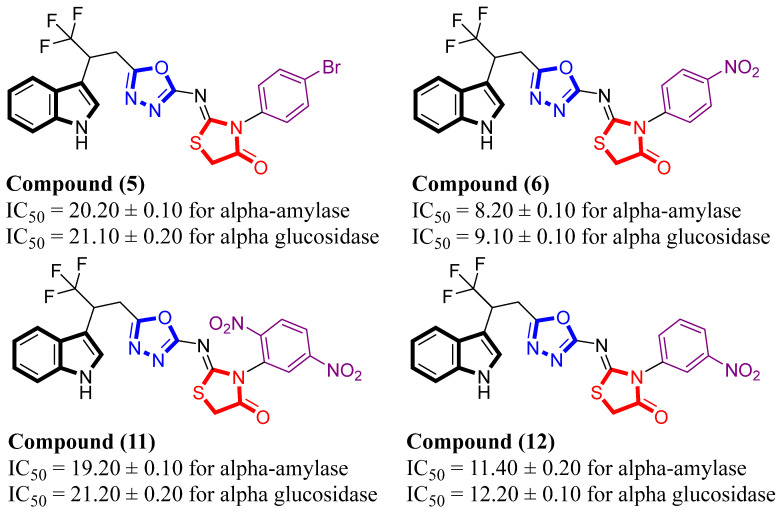
Inhibitory activity of derivatives-**5**, **6**, **11** and **12**.

Compound-**5**, having *p*-bromo, demonstrated an inhibition action of 20.20 ± 0.10 µM against alpha-amylase and 21.10 ± 0.20 µM against alpha-glucosidase. This might be due to the large atomic size of bromine, which causes stearic hindrance and resists the interaction of ligands with the targeted enzyme resulting in minimum inhibition action. Compound-**6**, which has *p*-nitro, presented an inhibition rate of 8.20 ± 0.10 µM against alpha-amylase, and in the case of alpha-glucosidase, its observed IC_50_ was 9.10 ± 0.10 µM. This might be due to the favorable position of the nitro group, which has the capability of hydrogen bond formation. Compound-**11**, having *o, m*-nitro, displayed an inhibition rate of 19.20 ± 0.10 µM and 21.20 ± 0.20 µM against the α-amylase enzyme and the α-glucosidase enzyme, respectively. This might be due to the electron-withdrawing nature of the nitro group, which withdraws electrons from the amino acids of target enzymes and binds them through different interactions. Compound-**12**, having *m*-nitro, showed an IC_50_ value of 11.40 ± 0.20 µM against α-amylase and 12.2 2 ± 0.20 µM against α-glucosidase. As the number of nitro groups is reduced, an increase in activity can be seen. Inhibitory activity of derivatives-**5**, **6**, **11** and **12** is given in Figure 4.

Compound-**7**, which holds a *p*-fluorine atom, demonstrated an exceptional level of activity of 5.60 ± 0.20 µM and 6.60 ± 0.10 µM. As fluorine atoms have a strong electronegative character along with the presence of a lone pair, a fluorine atom has the ability to activate the ring. Furthermore, it can also engage the enzyme via hydrogen bonding by using its lone pair of electrons.

**Figure 5:**
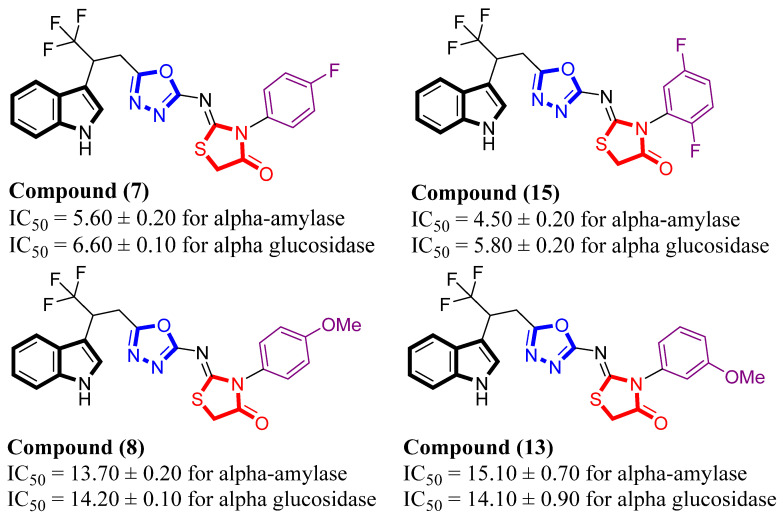
Inhibitory activity of derivatives-**7**, **8**, **13** and **15**.

Compound-**15**, having a fluorine atom at the *ortho* and *meta* positions, is the lead compound of the series, with the maximum inhibition action against amylase (an IC_50_ value of 4.50 ± 0.20 µM) and an IC_50_ value of 5.80 ± 0.80 µM against glucosidase. Standard drug acarbose was used for comparison, having IC_50_ values of 8.50 ± 0.10 µM and 9.30 ± 0.30 µM. Next, we have *p*-methoxy in analogue-**8**, with an IC_50_ value of 13.70 ± 0.20 µM against alpha-amylase and 14.20 ± 0.20 µM against alpha-glucosidase. As the methoxy group is stable due to hyperconjugation, it resists interaction with the targeted enzyme. Compound-**13**, having the *m*-methoxy group, showed IC_50_ values of 15.10 ± 0.70 µM and 14.10 ± 0.90 µM against both enzymes. Inhibitory activity of derivatives-**7**, **8**, **13** and **15** is given in Figure 5.

**Figure 6:**
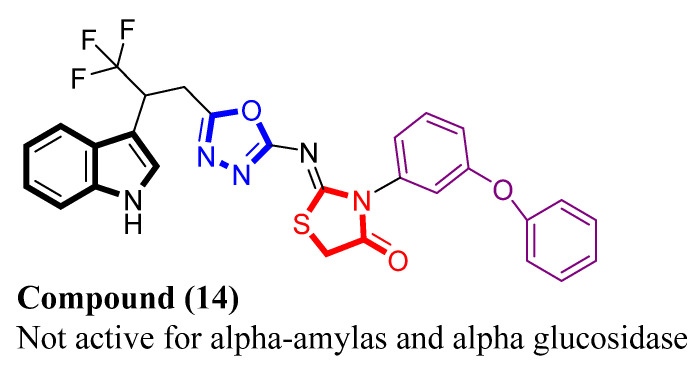
Inhibitory activity of derivative-**14**.

Compound-**14**, having benzo-oxy substitution, showed a non-active nature against both enzymes. This might be due to the bulkiness of the phenyl ring, and the ligand molecule was found to be unable to bind with the targeted protein. Inhibitory activity of derivatives-**14** is given in Figure 6.

### 2.3. Molecular Docking Study

**Figure 7:**
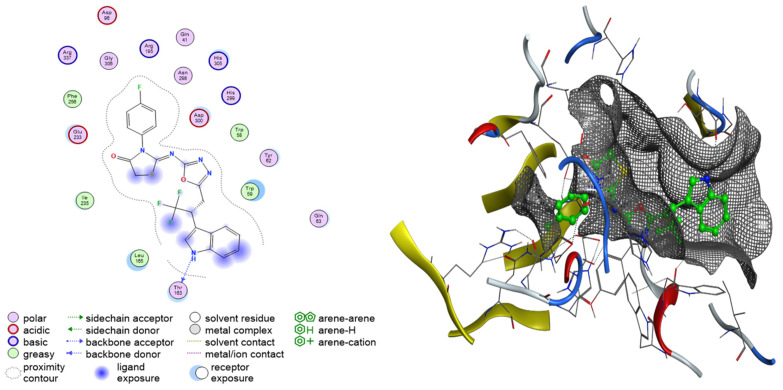
PLI profile of potent analog-**7** against alpha-amylase.

**Figure 8:**
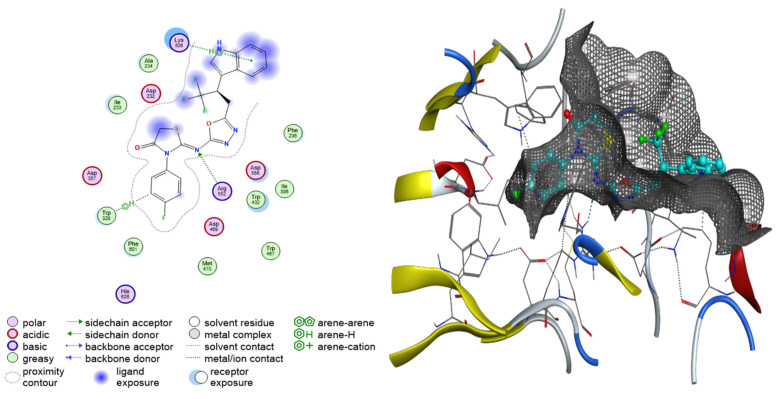
PLI profile of potent analog **7** against alpha-glucosidase.

**Figure 9:**
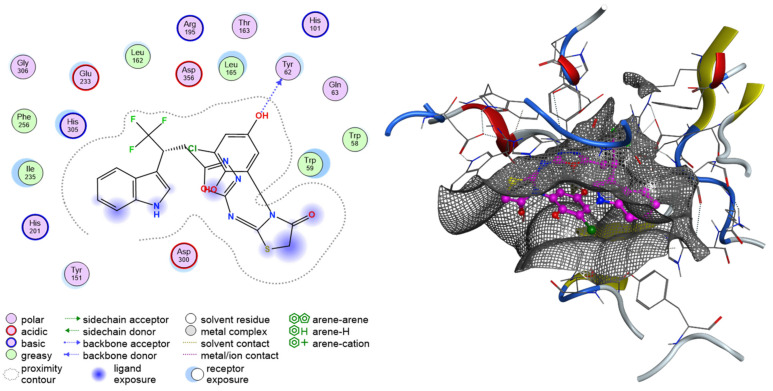
PLI profile of potent analog-**9** against alpha-amylase.

**Figure 10:**
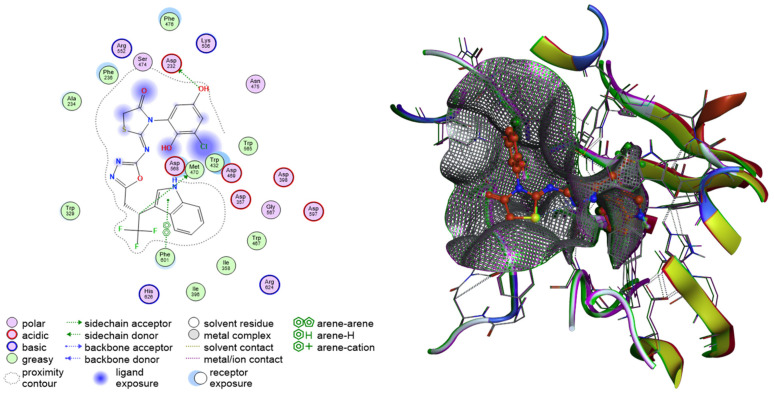
PLI profile of potent analog **9** against alpha-glucosidase.

**Figure 11:**
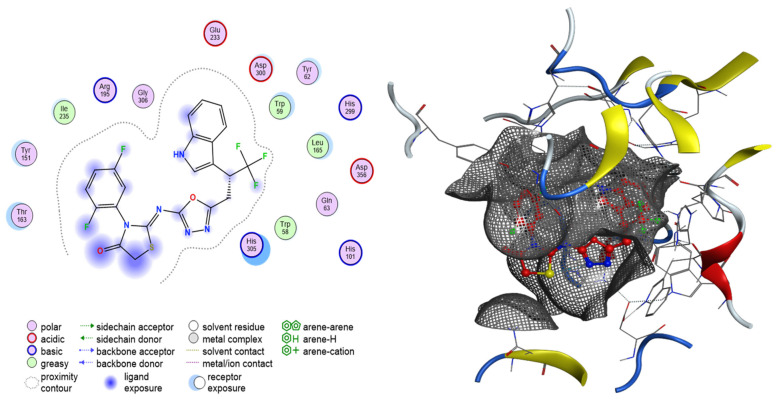
PLI profile of potent analog **15** against alpha-amylase.

**Figure 12:**
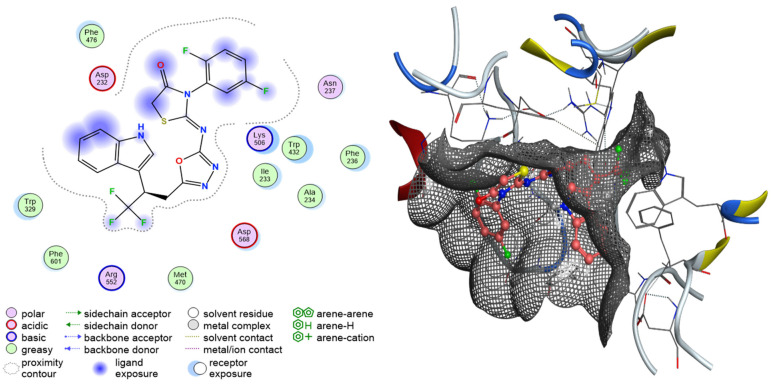
PLI profile of potent analog-**15** against alpha-glucosidase.

To ensure the inhibiting mechanism of potent ligands, a molecular docking study was performed. This study significantly contributes to understanding the biological profiles of molecules. We have performed a molecular docking investigation on potent compounds which were identified as leading inhibitors in biological assessment. We retrieved the protein molecules from the RCSB Protein Data Bank (PDB) using the specific identifier codes 1b2y for α-amylase and 3w37 for α-glucosidase. Both proteins were prepared for docking by the removal of co-crystallized ligand and water molecules. Polar hydrogen was also added. Different charges, including Kollman and Gasteiger, were also added. Ligand molecules were also prepared, and their energy was minimized using the tool MOE to find out the most stable conformation. Potent ligand molecules were docked at the active site of target enzymes by specifying the dummies. The results of the docking investigation disclosed that ligand molecules bind with the enzymes at the active site via several interactions. The binding site of an enzyme is engaged via the pi-system of the ligand, which interacts with the pi- system of the enzyme through pi–cation and pi–pi interactions. Along with these bonds, some other types of interactions were also exhibited by the potent compounds (Figure 7, Figure 8, Figure 9, Figure 10, Figure 11 and Figure 12). The docking score observed for compound-**7** was −9.5, while for compounds **9** and **15** the score was 10.4 and 9.1 respectively. Our research group has previously conducted molecular docking studies on various moieties against different proteins [35,36,37,38], and we applied a similar methodology in this research. The interactions between these ligands and the amino acids of the target proteins are illustrated as Protein–Ligand Interaction (PLI) profile in Figure 7, Figure 8, Figure 9, Figure 10, Figure 11 and Figure 12. The binding interactions of these compounds and their docking score are tabulated as Table 3.

Analog-**7** was docked against alpha-amylase, and different interactions were observed between the analog and amino acids on the active site of the enzyme. These interactions engage the amino acids of the enzyme, and thus the substrate molecule cannot bind with the active site to form an enzyme–substrate complex. The Protein–Ligand Interaction profile of the analog includes a Polar-Side chain acceptor, Pi–Pi bonds and Pi–H interactions with the amino acids THR-163, LEU-165, ILE-235, GLU-233, PHE-256, ARG-337, GLY-337, ASP-96, ARG-195, ASN-298, GLN-41, HIS-305, HIS-299, ASP-300, TRP-58, TYR-62, TRP-59 and GLN-63. The calculated docking score for these interactions was found to be −9.5. The PLI profile of analog-**7** against alpha-amylase is illustrated in Figure 7.

The PLI profile of analog-**7** was also studied against alpha-glucosidase, and the amino acids PHE-601, TRP-329, ASP-357, ILE-233, ALA-234, ASP-232, LYS-506, PHE-236, ASP-568, ILE-396, TRP-432, ARG-552, ASP-469, TRP-467, MET-470 and HIS-626 were found to interact with the analog. The Polar-Side chain acceptor, Pi–Pi bonds and Pi–H interactions were observed in the molecular docking study. Docking simulations yielded a score of −10.73, indicating strong binding interactions. The PLI profile of analog **7** against alpha-glucosidase is illustrated in Figure 8.

The Protein–Ligand Interaction (PLI) profile of analog-**9** was investigated against alpha-amylase, revealing key interactions with amino acids TYR-151, HIS-201, ILE-235, PHE-256c, GLY-306, HIS-305, GLU-233, LEU-162, ASP-356, ARG-195, LEU-165, THR-163, TYR-62, HIS-101, GLN-63, TRP-58 and TRP-59. The molecular docking analysis identified a Polar-Side chain acceptor, Pi–Pi bonds and Pi–H interactions, with a docking score of −10.4, indicating strong binding affinity. The PLI profile of analog-**9** against alpha-amylase is depicted in Figure 9.

Analog-**9**, docked against alpha-glucosidase, showed strong interactions with 24 amino acids, including TRP-329, ALA-234, PHE-236, ARG-552, SER-474, ASP-232, PHE-476, LYS-506, ASN-475, ASP-568, MET-470, TRP-432, ASP-469, TRP-565, ASP-357, ASP-398, GLY-567, ASP-597, TRP-467, ILE-358, ARG-624, ILE-396, PHE-601 and HIS-626, through the Polar-Side chain acceptor, Pi–Pi bonds and Pi–H interactions. This binding blocks substrate access to the active site, with a docking score of −12.04. The PLI profile of analog-**9** against alpha-glucosidase is illustrated in Figure 10.

Analog **15** exhibited strong binding to alpha-amylase, interacting with 16 amino acids, THR-163, TYR-151, ILE-235, ARG-195, GLY-306, GLU-233, ASP-300, TRP-59, TYR-62, HIS-299, LEU-165, ASP-356, GLN-63, HIS-101, TRP-58 and HIS-305, through the Polar-Side chain acceptor, Pi–Pi bonds and Pi–H interactions. The docking score of −9.1 indicates high affinity. The PLI profile of analog-**15** is shown in Figure 11.

Analog-**15** demonstrated potent binding to alpha-glucosidase, engaging 13 key amino acids. The amino acids TRP-329, ASP-232, PHE-476, ASN-237, LYS-506, TRP-432, PHE-236, ALA-234, ILE-233, ASP-568, MET-470, ARG-552 and PHE-601 bound with analog-**15** through favorable interactions, including the Polar-side chain acceptor, Pi–Pi bonds and Pi–H interactions. The docking score of −9.8 indicates a strong binding affinity, highlighting Analog 15’s potential as an alpha-glucosidase inhibitor. The PLI profile of analog-**15** is shown in Figure 12.

**Figure 13:**
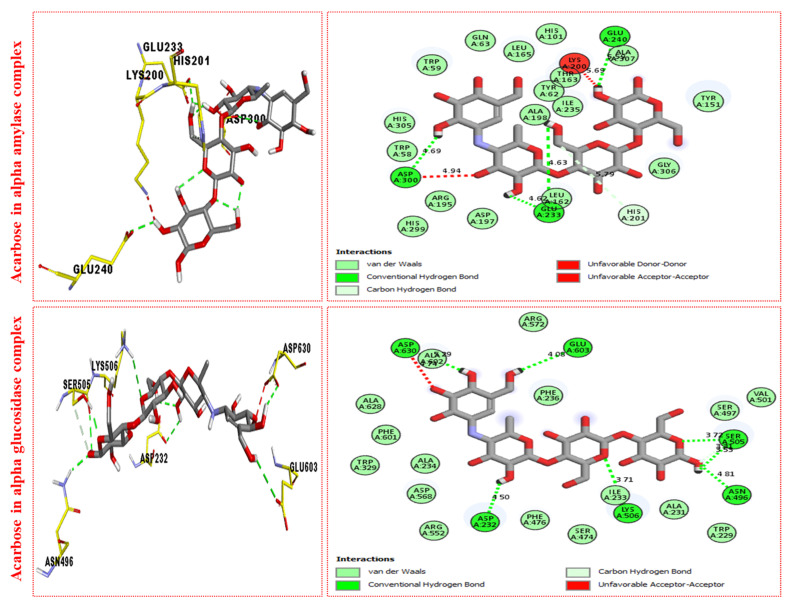
PLI profile of acarbose against alpha-amylase and alpha-glucosidase.

Moreover, the standard drug acarbose was also docked against both enzymes and is illustrated in Figure 13. Different interactions were observed in both cases, but the lesser interactions show that the potential of acarbose to inhibit both enzymes is lower than the potent compounds of the current research work.

The molecular docking study of potent compounds against both enzymes revealed the inhibitory potential as expressed in biological activity evaluation. The different binding interactions with target enzymes strengthen the biological profile of the compounds in view of the docking study.

### 2.4. ADMET Analysis

In the realm of drug design, minimum side effects with great effectiveness are in high demand. To evaluate the drug attributes of our potent compounds, ADMET analysis was also conducted. Table 4, given below, shows detailed information on the drug-like characteristics of the lead compounds. Potent compounds were analyzed for their drug-likeness via ADMET analysis and were found safer therapeutic agents.

The drug-likeness analysis of analogs **7**, **8** and **15** show that these compounds comply on the standard drug-likeness rules such as the Lipinski rule of five. The molecular weight of analog **7** was calculated as 489.45 g/mol. As the molecular mass of analog **7** is lower than 500 g/mol, it reflects pharmacokinetic drug potential according to the Lipinski rule. The number of hydrogen bond acceptors in analogs **7**, **8** and **15** is 9, 10 and 10, respectively, while the number of hydrogen bond acceptors is 1, 3 and 1, respectively, which also falls within the range of acceptable drug-likeness. The bioavailability score of analogs **7**, **8** and **15** is 0.55, 0.55 and 0.17, respectively. This score represents their good absorption and distribution within the body, as well as potential efficacy. Some other pharmacokinetic attributes of these potent analogs were also evaluated and are presented in Table 4.

## 3. Experimental

### 3.1. General Information

^1^H and ^13^C NMR spectra were recorded in DMSO on a Bruker AM spectrometer (500 MHz, frequency for ^13^C NMR). Chemical shifts (*δ*) are reported in parts per million (ppm) calibrated from TMS (0 ppm) as an internal standard for ^1^H NMR, and solvent (shift) for ^13^C NMR.Coupling constant (J) values are expressed in Hz. The reaction course and purity of the synthesized compounds were monitored by TLC in a mobile phase of mixture containing ethyl acetate and n-hexane in 1:3 using TLC plates. Melting points were measured on Buchi M-560 melting point apparatus.

### 3.2. Synthesis of Hybrid Indole-Oxadiazole Linked Thiazolidinone Derivatives *(****1****–****15****)*

#### 3.2.1. Synthesis of Oxadiazole Based Indole as First Intermediate

To synthesize indole-derived oxadiazole-bearing thiazolidinone moiety, 1 mmol of indole-bearing ester was mixed with 1 mmol of semicarbazide. These reactants were mixed in 15 mL ethanol under reflux for 7 h. The reaction mixture was kept under constant stirring at a temperature of 145 °C. The single spot TLC confirmed the synthesis of oxadiazole-based indole as first intermediate. For TLC, a mixture of ethyl acetate and n-hexane was used in 2:3. This intermediate was synthesized with a good yield of 74%. For purification, the intermediate was washed with n-hexane. The solvent used for washing was then evaporated at reduced pressure, and the fine powdered product was collected.

#### 3.2.2. Synthesis of Indole Derived Oxadiazole Bearing Thiourea Derivatives

In this step, 1 mmol of oxadiazole-based indole was treated with 1 mmol of differently substituted isothiocyanate. This reaction was carried out in 15 mL ethanol. To catalyze the reaction, three drops of Et_3_N were also added to the reaction mixture. After 5 h of continuous stirring under reflux conditions, indole-derived oxadiazole-bearing thiourea derivatives were produced. The reaction was carried out at a temperature of 140 °C. the percentage yield of these derivatives was found to range between 63 and 81%. After washing and drying, all the derivatives were collected as fine powder.

#### 3.2.3. Synthesis of Hybrid Indole-Oxadiazole Linked Thiazolidinone Derivatives

Lastly, 1 mmol of each indole-derived oxadiazole-bearing thiourea derivative was treated with 1 mmol of chloroacetic acid in the presence of acetic acid as catalyst (three drops). The reaction mixture was stirred in 15 mL of ethanol for 12 h under reflux conditions. The desired hybrid indole–oxadiazole-linked thiazolidinone derivatives were collected after a single spot TLC. All the derivatives were washed with n-hexane, and the solvent was evaporated at reduced pressure.

Spectral analysis, biological activity assay and molecular docking assay is provided in Supplementary information.

## 4. Conclusions

In the current research study, a library of hybrid indole–oxadiazole-linked thiazolidinone derivatives (**1**–**15**) were designed and synthesized via a multistep synthetic route. The synthesis of these compounds was achieved by treating an indole derivative with semicarbazide. The resultant product was further reacted with isothiocyanate with different substitutions, followed by treating the resultant product with chloroacetic acid. These compounds were structurally confirmed via spectroscopic techniques (^13^C-NMR and ^1^H-NMR). A biological activity evaluation of all the compounds against α-amylase and α-glucosidase was carried out. In this analysis, analog **15**, **9** and **7** emerged as the excellent enzyme inhibitors, with inhibition concentration (IC_50_) values of 4.50 ± 0.20, 7.30 ± 0.20 and 5.60 ± 0.20 µM for α-amylase and 5.80 ± 0.80 7.60 ± 0.20 and 5.60 ± 0.20 µM for α-glucosidase, respectively. For the comparative study, acarbose (IC_50_ = 8.50 ± 0.10 for α-amylase and 9.30 ± 0.30 for α-glucosidase) was used as standard. To validate the inhibitory actions of potent analogs, a molecular docking study was conducted which provided insight into the binding interactions of these compounds against both enzymes. To determine the drug-likeness of reported potent analogs, an ADMET investigation was also carried out. This research introduces novel heterocyclic compounds as promising anti-diabetic agents with significant potential for future therapeutic applications.

## Figures and Tables

**Table 1:** Percentage yield of all the novel hybrid indole–oxadiazole linked thiazolidinone derivatives (**1**–**15**).

Compound	Percentage (%) Yield	Compound	Percentage (%) Yield
**1**	63	**9**	72
**2**	65	**10**	66
**3**	73	**11**	78
**4**	69	**12**	81
**5**	78	**13**	69
**6**	74	**14**	77
**7**	70	**15**	82
**8**	72		

**Table 2:** Inhibition concentration (IC_50_) of hybrid indole–oxadiazole-linked thiazolidinone derivatives against alpha-amylase and alpha-glucosidase in comparison to standard acarbose.

S/No	R_1_	Alpha Amylase IC_50_ (µM)	Alpha Glucosidase IC_50_ (µM)
1	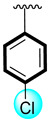	13.10 ± 0.40	12.30 ± 0.10
2	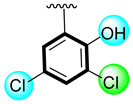	8.20 ± 0.10	9.10 ± 0.30
3	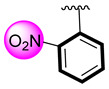	15.30 ± 0.20	14.50 ± 0.20
4	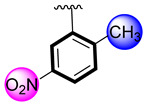	12.10 ± 0.10	12.70 ± 0.30
5	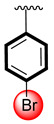	20.20 ± 0.10	21.10 ± 0.20
6	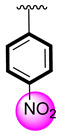	8.20 ± 0.10	9.10 ± 0.10
7	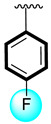	5.60 ± 0.20	6.60 ± 0.10
8	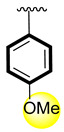	13.70 ± 0.20	14.20 ± 0.10
9	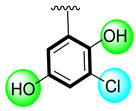	7.30 ± 0.20	7.60 ± 0.20
10	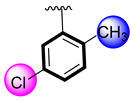	10.20 ± 0.10	12.20 ± 0.50
11	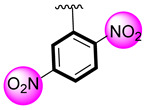	19.20 ± 0.10	21.20 ± 0.20
12	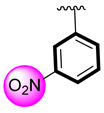	11.40 ± 0.20	12.20 ± 0.10
13	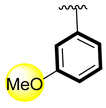	15.10 ± 0.70	14.10 ± 0.90
14	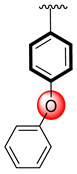	N.A	N.A
15	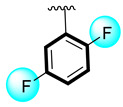	4.50 ± 0.20	5.80 ± 0.20
Standard drug Acarbose		8.50 ± 0.10	9.30 ± 0.30

**Table 3:** Binding interactions of potent compounds and their docking score.

Active Analogs	Receptors	Types of Interactions	Docking Score
Analog-**7** in alpha amylase	THR-163	Polar-Side chain acceptor Pi-Pi bonds Pi-H	−9.5
	LEU-165		
	ILE-235		
	GLU-233		
	PHE-256		
	ARG-337		
	GLY-337		
	ASP-96		
	ARG-195		
	ASN-298		
	GLN-41		
	HIS-305		
	HIS-299		
	ASP-300		
	TRP-58		
	TYR-62		
	TRP-59		
	GLN-63		
Analog-**7** in alpha glucosidase	PHE-601	Polar-Side chain acceptor Pi-Pi bonds Pi-H	−10.73
	TRP-329		
	ASP-357		
	ILE-233		
	ALA-234		
	ASP-232		
	LYS-506		
	PHE-236		
	ASP-568		
	ILE-396		
	TRP-432		
	ARG-552		
	ASP-469		
	TRP-467		
	MET-470		
	HIS-626		
Analog-**9** in alpha amylase	TYR-151	Polar-Side chain acceptor Pi-Pi bonds Pi-H	−10.4
	HIS-201		
	ILE-235		
	PHE-256		
	GLY-306		
	HIS-305		
	GLU-233		
	LEU-162		
	ASP-356		
	ARG-195		
	LEU-165		
	THR-163		
	TYR-62		
	HIS-101		
	GLN-63		
	TRP-58		
	TRP-59		
Analog-**9** in alpha glucosidase	TRP-329	Polar-Side chain acceptor Pi-Pi bonds Pi-H	−9.04
	ALA-234		
	PHE-236		
	ARG-552		
	SER-474		
	ASP-232		
	PHE-476		
	LYS-506		
	ASN-475		
	ASP-568		
	MET-470		
	TRP-432		
	ASP-469		
	TRP-565		
	ASP-357		
	ASP-398		
	GLY-567		
	ASP-597		
	TRP-467		
	ILE-358		
	ARG-624		
	ILE-396		
	PHE-601		
	HIS-626		
Analog-**15** in alpha amylase	THR-163	Polar-Side chain acceptor Pi-Pi bonds Pi-H	−9.1
	TYR-151		
	ILE-235		
	ARG-195		
	GLY-306		
	GLU-233		
	ASP-300		
	TRP-59		
	TYR-62		
	HIS-299		
	LEU-165		
	ASP-356		
	GLN-63		
	HIS-101		
	TRP-58		
	HIS-305		
Analog-**15** in alpha glucosidase	TRP-329	Polar-Side chain acceptor Pi-Pi bonds Pi-H	−9.8
	ASP-232		
	PHE-476		
	ASN-237		
	LYS-506		
	TRP-432		
	PHE-236		
	ALA-234		
	ILE-233		
	ASP-568		
	MET-470		
	ARG-552		
	PHE-601		

**Table 4:** ADMET analysis of potent analogs **7, 8** and **15**.

Compound	Mol.wt (g/mol)	H-Bond Acceptors	H-Bond Donor	Log S (ESOL)	Solubility	Log *P*_o/w_ (ILOGP)	Log *K*_p_ (Skin Permeation) cm/s	Lipinski	Ghose	Veber	Egan	Muegge	Bioavailability Score	PAINS	Brenk	Leadlikeness	Synthetic Accessibility
7	489.45	9	1	−6.19	3.16 × 10^−4^ mg/mL; 6.46 × 10^−7^ mol/L	3.36	−5.59	Yes; 0 violation	No; 2 Violation; MW > 480, WLOGP > 5.6	Yes	No; 1 Violation; WLOGP > 5.88	No; 1 Violation; XLOGP3 > 5	0.55	0 alert	1 alert: imine-1	No; 2 violations; MW > 350, Rotors > 7, XLOGP3 > 3.5	4.41
9	537.90	10	3	−6.35	2.42 × 10^−4^ mg/mL; 4.50 × 10^−7^ mol/L	2.06	−6.02	Yes; 1 violation: MW > 500	No; 3 Violations; MW > 480, WLOGP > 5.6, MR> 130	No; 1 Violation: TPSA > 140	No; 2 Violation: WLOGP > 5.88TPSA > 131.6	No; 2 Violation: XLOGP3>5, TPSA > 150	0.55	0 alert	2 alert: hyroquinone, imine-1,	No; 2 violations; MW > 350, XLOGP3 > 3.5,	4.53
24	507.44	10	1	−6.35	2.26 × 10^−3^ mg/mL; 3.81 × 10^−6^ mol/L	2.78	−5.63	No; 2 violation; MW > 500 MILOP > 4.15	No; 2 Violations; MW > 480, WLOGP > 5.6	Yes	No; 1 Violation: WLOGP > 5.88	No; 2 Violation; XLOGP3 > 5	0.17	0 alert	1 alert: imine-1	No; 2 violation; MW > 350, XLOGP3 > 3.5	4.46

## Data Availability

Data is contained within the article and Supplementary Material.

## Supplementary Materials

The following supporting information can be downloaded at: https://www.mdpi.com/article/10.3390/ph17111428/s1, Figure S1: 1HNMR for the compound 1 3-(4-chlorophenyl)-2-((5-(3,3,3-trifluoro-2-(1H-indol-3-yl)propyl)-1,3,4-oxadiazol-2-yl)imino)thiazolidin-4-one); Figure S2: 1HNMR for the compound 2 3-(4-nitrophenyl)-2-((5-(3,3,3-trifluoro-2-(1H-indol-3-yl)propyl)-1,3,4-oxadiazol-2-yl)imino)thiazolidin-4-one); Figure S3: 1HNMR for the compound 12 3-(3-nitrophenyl)-2-((5-(3,3,3-trifluoro-2-(1H-indol-3-yl)propyl)-1,3,4-oxadiazol-2-yl)imino)thiazolidin-4-one); Figure S4: 1HNMR for the compound 15 3-(2,5-difluorophenyl)-2-((5-(3,3,3-trifluoro-2-(1H-indol-3-yl)propyl)-1,3,4-oxadiazol-2-yl)imino)thiazolidin-4-one); Figure S5: 13CNMR for the compound 1 3-(4-chlorophenyl)-2-((5-(3,3,3-trifluoro-2-(1H-indol-3-yl)propyl)-1,3,4-oxadiazol-2-yl)imino)thiazolidin-4-one); Figure S6: 13CNMR for the compound 6 3-(4-nitrophenyl)-2-((5-(3,3,3-trifluoro-2-(1H-indol-3-yl)propyl)-1,3,4-oxadiazol-2-yl)imino)thiazolidin-4-one); Figure S7: 13CNMR for the compound 12 3-(3-nitrophenyl)-2-((5-(3,3,3-trifluoro-2-(1H-indol-3-yl)propyl)-1,3,4-oxadiazol-2-yl)imino)thiazolidin-4-one); Figure S8: 13CNMR for the compound 15 3-(2,5-difluorophenyl)-2-((5-(3,3,3-trifluoro-2-(1H-indol-3-yl)propyl)-1,3,4-oxadiazol-2-yl)imino)thiazolidin-4-one); Figure S9: 13CNMR for the compound 7 3-(4-fluorophenyl)-2-((5-(3,3,3-trifluoro-2-(1H-indol-3-yl)propyl)-1,3,4-oxadiazol-2-yl)imino)thiazolidin-4-one); Figure S10: 13CNMR for the compound 5 3-(4-bromophenyl)-2-((5-(3,3,3-trifluoro-2-(1H-indol-3-yl)propyl)-1,3,4-oxadiazol-2-yl)imino)thiazolidin-4-one). References [39,40,41] are cited in the supplementary materials.
