# Supplementary material for: Synthesis, Characterizations, Anti-Diabetic and Molecular Modeling Approaches of Hybrid Indole-Oxadiazole Linked Thiazolidinone Derivatives
Source: Pharmaceuticals (Basel). 2024 Oct 24;17(11):1428. doi: 10.3390/ph17111428 (PMC11597374; doi:10.3390/ph17111428)
Supplement: Supplementary file 1 [file pharmaceuticals-17-01428-s001.zip › pharmaceuticals-3223166-supplementary.pdf]

Synthesis, characterizations, anti-diabetic and molecular modeling approaches of hybrid indole-oxadiazole linked thiazolidinone derivatives

Shoaib Khan[1*], Tayyiaba Iqbal[1], Rafaqat Hussain[2], Yousaf Khan[3], Zanib Fiaz[1], Fazal Rahim[4], Hany W. Darwish[5]

[1]Department of Chemistry, Abbottabad University of Science and Technology (AUST) Abbottabad-22500, Pakistan.
[2]College of Biology, Hunan University Changsha, Hunan-410082, P. R. China
[3]Department of Chemistry, COMSATS University Islamabad 45550, Islamabad Pakistan
[4]Department of Chemistry, Hazara University Mansehra, Pakistan, 21300, Pakistan
[5]Department of Pharmaceutical Chemistry, College of Pharmacy, King Saud University, P.O. Box 2457, Riyadh 11451, Saudi Arabia.

[*]Corresponding authors: *shoaibkhanswati@gmail.com (S. Khan*)

S.1. Spectral Analysis

3-(4-Chlorophenyl)-2-((5-(3,3,3-trifluoro-2-(1H-indol-3-yl)propyl)-1,3,4-oxadiazol-2-yl)imino)thiazolidin-4-one) 1 (Compound 1)

^{1}H NMR (600MHz, DMSO-*d$_6$*): δ 12.07 (s, 1H, H-Indole), 8.83 (dd, *J* = 7.26, 2.43 Hz, 1H, H-Indole), 8.56 (m, *J* = 6.5-7.5 Hz, 1H, H-Indole), 8.27 (d, *J* = 7.56 Hz, 2H, Ar-H), 7.84 (dd, *J* = 7.41, 2.81 Hz, 1H, H-Indole), 7.73 (d, *J* = 7.41 Hz, 2H, Ar-H), 7.49 (s, 1H, H-Indole), 7.29 (m, *J* = 6.3-7.3 Hz, 1H, H-Indole), 3.62 (t, *J* = 4.5 Hz, 1H, H-Aliphatic), 2.18 (s, 2H, H-Thiazolidinone), 2.02 (d, *J* = 4.3 Hz, 2H, H-Aliphatic); ^{13}C NMR (125 MHz, DMSO-d$_6$): δ 181.3, 164.0, 153.5, 151.8, 151.3, 151.1, 143.5, 141.0, 139.9, 136.5, 134.3, 130.7, 126.6, 124.5, 123.3, 121.0, 50.2, 34.4, 25.2; HREI MS :*m/z* calcd for $C_{22}H_{15}ClF_3N_5O_2S$ $[M]^+$ 505.90 Found 505.38

3-(3,5-Dichloro-2-hydroxyphenyl)-2-((5-(3,3,3-trifluoro-2-(1H-indol-3-yl)propyl)-1,3,4-oxadiazol-2-yl)imino)thiazolidin-4-one) (Compound 2)

^{1}H NMR (600MHz, DMSO-*d$_6$*): δ 12.73 (s, 1H, H-Indole), 9.53 (s, 1H, H-OH), 7.55 (dd, *J* = 7.26, 2.43 Hz, 1H, H-Indole), 7.53 (s, 1H, Ar-H), 7.37 (dd, *J* = 7.41, 2.81 Hz, 1H, H-Indole), 7.14 (s, 1H, H-Indole), 7.12 (s, 1H, Ar-H), 7.10 (m, *J* = 6.5-7.5 Hz, 1H, H-Indole), 7.07 (m, *J* = 6.3-7.3 Hz, 1H, H-Indole) 4.27 (t, *J* = 4.5 Hz, 1H, H-Aliphatic), 3.13 (s, 2H, H-Thiazolidinone), 2.25 (d, *J* = 4.3 Hz, 2H, H-Aliphatic); ^{13}C NMR (125 MHz, DMSO-d$_6$): δ 172.3, 163.7, 163.5, 155.7, 147.3, 136.2, 128.5, 127.5, 127.4, 126.4, 125.5, 123.5, 121.5, 121.3, 119.5, 118.5, 117.5, 113.5, 111.4, 50.4, 33.7, 19.3; HREI MS :*m/z* calcd for $C_{22}H_{14}Cl_2F_3N_5O_3S$ $[M]^+$ 556.34 Found 556.11

***3-(2-Nitrophenyl)-2-((5-(3,3,3-trifluoro-2-(1H-indol-3-yl)propyl)-1,3,4-oxadiazol-2-yl)imino)thiazolidin-4-one) (*Compound 3)

^{1}H NMR (600MHz, DMSO-*d6*): *δ* 10.73 (s, 1H, H-Indole), 8.37 (dd, *J* = 7.41, 2.81 Hz, 1H, Ar-H), 7.95 (dd, *J* = 7.26, 2.43 Hz, 1H, Ar-H) 7.85 (m, *J* = 6.5-7.5 Hz, 1H, Ar-H), 7.75 (m, *J* = 6.3-7.3 Hz, 1H, Ar-H), 7.53 (dd, *J* = 7.26, 2.43 Hz, 1H, H-Indole), 7.37 (dd, *J* = 7.41, 2.81 Hz, 1H, H-Indole), 7.14 (s, 1H, H-Indole), 7.11 (m, *J* = 6.5-7.5 Hz, 1H, H-Indole), 7.07 (m, *J* = 6.3-7.3 Hz, 1H, H-Indole) 4.27 (t, *J* = 4.5 Hz, 1H, H-Aliphatic), 2.99 (s, 2H, H-Thiazolidinone), 2.25 (d, *J* = 4.3 Hz, 2H, H-Aliphatic); ^{13}C NMR (125 MHz, DMSO-d$_6$): *δ* 172.3, 163.7, 163.5, 155.7, 142.1, 136.2, 135.3, 135.1, 128.1, 127.4, 125.5, 125.3, 123.5, 122.3, 121.3, 119.5, 118.5, 113.5, 111.4, 50.4, 33.7, 19.3; HREI MS :*m/z* calcd for $C_{22}H_{15}F_3N_6O_4S$ [M]$^+$ 516.46 Found 516.12

***3-(2-Methyl-5-nitrophenyl)-2-((5-(3,3,3-trifluoro-2-(1H-indol-3-yl)propyl)-1,3,4-oxadiazol-2-yl)imino)thiazolidin-4-one) (*Compound 4)

^{1}H NMR (600MHz, DMSO-*d6*): *δ* 11.93 (s, 1H, H-Indole), 8.52 (s, 1H, Ar-H), 7.94 (d, *J* = 7.41 Hz, 1H, Ar-H), 7.55 (dd, *J* = 7.26, 2.43 Hz, 1H, H-Indole), 7.54 (d, *J* = 7.56 Hz, 1H, Ar-H), 7.37 (dd, *J* = 7.41, 2.81 Hz, 1H, H-Indole), 7.14 (s, 1H, H-Indole), 7.10 (m, *J* = 6.5-7.5 Hz, 1H, H-Indole), 7.07 (m, *J* = 6.3-7.3 Hz, 1H, H-Indole), 3.99 (t, *J* = 4.5 Hz, 1H, H-Aliphatic) 3.21 (s, 2H, H-Thiazolidinone), 1.25 (d, *J* = 4.3 Hz, 2H, H-Aliphatic), 2.15 (s, 3H, H-Aliphatic); ^{13}C NMR (125 MHz, DMSO-d$_6$): *δ* 172.3, 163.7, 163.5, 155.7, 145.3, 140.3, 139.5, 136.2, 131.5, 130.4, 128.5, 127.5, 123.5, 121.5, 119.5, 118.5, 114.7, 113.5, 111.4, 50.4, 33.7, 19.3, 17.5; HREI MS :*m/z* calcd for $C_{23}H_{17}F_3N_6O_4S$ [M]$^+$ 530.48 Found 530.11

***3-(4-Bromophenyl)-2-((5-(3,3,3-trifluoro-2-(1H-indol-3-yl)propyl)-1,3,4-oxadiazol-2-yl)imino)thiazolidin-4-one) (*Compound 5)

^{1}H NMR (600MHz, DMSO-*d6*): *δ* 11.98 (s, 1H, H-Indole), 7.67 (d, *J* = 7.41 Hz, 2H, Ar-H), 7.64 (d, *J* = 7.56 Hz, 2H, Ar-H), 7.55 (dd, *J* = 7.26, 2.43 Hz, 1H, H-Indole), 7.37 (dd, *J* = 7.41, 2.81 Hz, 1H, H-Indole), 7.14 (s, 1H, H-Indole), 7.10 (m, *J* = 6.5-7.5 Hz, 1H, H-Indole), 7.07 (m, *J* = 6.3-7.3 Hz, 1H, H-Indole), 4.28 (t, *J* = 4.5 Hz, 1H, H-Aliphatic), 2.98 (s, 2H, H-Thiazolidinone), 2.65 (d, *J* = 4.3 Hz, 2H, H-Aliphatic); ^{13}C NMR (125 MHz, DMSO-d$_6$): *δ* 178.2, 161.6, 150.8, 150.4, 150.3, 149.2, 141.5, 139.0, 137.9, 132.9, 129.2, 128.7, 125.0, 123.8, 122.3, 50.2, 33.5, 20.7; HREI MS :*m/z* calcd for $C_{22}H_{15}BrF_3N_5O_2S$ [M]$^+$ 550.35 Found 550.11

3-(4-Nitrophenyl)-2-((5-(3,3,3-trifluoro-2-(1H-indol-3-yl)propyl)-1,3,4-oxadiazol-2-yl)imino)thiazolidin-4-one) (Compound 6)

^{1}H NMR (600MHz, DMSO-*d6*): δ 12.24 (s, 1H, H-Indole), 8.36 (d, *J* = 7.41 Hz, 2H, Ar-H), 8.28 (d, *J* = 7.56 Hz, 2H, Ar-H), 8.07 (dd, *J* = 7.26, 2.43 Hz, 1H, H-Indole), 7.83 (dd, *J* = 7.41, 2.81 Hz, 1H, H-Indole), 7.07 (s, 1H, H-Indole), 6.94 (m, *J* = 6.5-7.5 Hz, 2H, H-Indole), 3.57 (t, *J* = 4.5 Hz, 1H, H-Aliphatic), 2.35 (s, 2H, H-Thiazolidinone), 1.92 (d, *J* = 4.3 Hz, 2H, H-Aliphatic); ^{13}C NMR (125 MHz, DMSO-d6): δ 172.1, 163.5, 162.1, 154.5, 152.5, 151.7, 137.3, 131.5, 129.3, 127.8, 122.8, 121.6, 121.3, 112.5, 111.2, 109.7, 104.1, 54.9, 33.7, 21.1; HREI MS :*m/z* calcd for $C_{22}H_{15}F_{3}N_{6}O_{4}S$ $[M]^{+}$ 516.46 Found 516.11

3-(4-Fluorophenyl)-2-((5-(3,3,3-trifluoro-2-(1H-indol-3-yl)propyl)-1,3,4-oxadiazol-2-yl)imino)thiazolidin-4-one) (Compound 7)

^{1}H NMR (600MHz, DMSO-*d6*): δ 10.73 (s, 1H, H-Indole), 7.55 (dd, *J* = 7.26, 2.43 Hz, 1H, H-Indole), 7.53 (d, *J* = 7.41 Hz, 2H, Ar-H), 7.45 (d, *J* = 7.56 Hz, 2H, Ar-H), 7.37 (dd, *J* = 7.41, 2.81 Hz, 1H, H-Indole), 7.14 (s, 1H, H-Indole), 7.10 (m, *J* = 6.5-7.5 Hz, 1H, H-Indole), 7.07 (m, *J* = 6.3-7.3 Hz, 1H, H-Indole), 4.08 (t, *J* = 4.5 Hz, 1H, H-Aliphatic), 2.95 (s, 2H, H-Thiazolidinone), 2.34 (d, *J* = 4.3 Hz, 2H, H-Aliphatic); ^{13}C NMR (125 MHz, DMSO-d6): δ 175.7, 161.3, 161.2, 160.2, 147.5, 141.5, 139.3, 137.8, 132.8, 128.6, 123.8, 122.3, 121.6, 120.6, 114.3, 113.8, 52.8, 35.1, 23.7; HREI MS :*m/z* calcd for $C_{22}H_{15}F_{4}N_{5}O_{2}S$ $[M]^{+}$ 489.45 Found 489.11

3-(4-Methoxyphenyl)-2-((5-(3,3,3-trifluoro-2-(1H-indol-3-yl)propyl)-1,3,4-oxadiazol-2-yl)imino)thiazolidin-4-one) (Compound 8)

^{1}H NMR (600MHz, DMSO-*d6*): δ 11.73 (s, 1H, H-Indole), 7.55 (dd, *J* = 7.26, 2.43 Hz, 1H, H-Indole), 7.37 (dd, *J* = 7.41, 2.81 Hz, 1H, H-Indole), 7.17 (d, *J* = 7.41 Hz, 2H, Ar-H), 7.14 (s, 1H, H-Indole), 7.12 (m, *J* = 6.5-7.5 Hz, 1H, H-Indole), 7.09 (d, *J* = 7.56 Hz, 2H, Ar-H), 7.07 (m, *J* = 6.3-7.3 Hz, 1H, H-Indole), 3.21 (t, *J* = 4.5 Hz, 1H, H-Aliphatic), 2.30 (s, 2H, H-Thiazolidinone), 1.85 (s, 3H, H-Aliphatic), 1.75 (d, *J* = 4.3 Hz, 2H, H-Aliphatic); ^{13}C NMR (125 MHz, DMSO-d6): δ 172.3, 163.7, 163.5, 158.5 155.7, 136.3, 129.6, 128.6, 128.5, 128.3, 127.5, 123.5, 121.5, 119.5, 118.5, 114.5, 114.3, 113.5, 111.4, 55.5, 50.4, 33.7, 19.3; HREI MS :*m/z* calcd for $C_{23}H_{18}F_{3}N_{5}O_{3}S$ $[M]^{+}$ 501.48 Found 501.11

3-(3-Chloro-2,5-dihydroxyphenyl)-2-((5-(3,3,3-trifluoro-2-(1H-indol-3-yl)propyl)-1,3,4-oxadiazol-2-yl)imino)thiazolidin-4-one) (Compound 9)

^{1}H NMR (600MHz, DMSO-*d6*): *δ* 11.84 (s, 1H, H-Indole), 9.54 (s, 1H, H-OH), 9.43 (S, 1H, H-OH), 7.55 (dd, *J* = 7.26, 2.43 Hz, 1H, H-Indole), 7.37 (dd, *J* = 7.41, 2.81 Hz, 1H, H-Indole), 7.14 (s, 1H, H-Indole), 7.12 (m, *J* = 6.5-7.5 Hz, 1H, H-Indole), 7.07 (m, *J* = 6.3-7.3 Hz, 1H, H-Indole), 7.05 (s, 1H, Ar-H), 7.03 (s, 1H, Ar-H), 4.27 (t, *J* = 4.5 Hz, 1H, H-Aliphatic), 3.56 (s, 2H, H-Thiazolidinone), 2.25 (d, *J* = 4.3 Hz, 2H, H-Aliphatic); ^{13}C NMR (125 MHz, DMSO-d6): *δ* 172.3, 163.7, 163.5, 158.5 155.7, 136.3, 129.6, 128.6, 128.5, 128.3, 127.5, 123.5, 121.5, 119.5, 118.5, 114.5, 114.3, 113.5, 111.4, 55.5, 50.4, 33.7, 19.3; HREI MS :*m/z* calcd for $C_{22}H_{15}ClF_3N_5O_4S$ $[M]^+$ 537.90 Found 537.41

3-(5-Chloro-2-methylphenyl)-2-((5-(3,3,3-trifluoro-2-(1H-indol-3-yl)propyl)-1,3,4-oxadiazol-2-yl)imino)thiazolidin-4-one) (Compound 10)

^{1}H NMR (600MHz, DMSO-*d6*): *δ* 12.03 (s, 1H, H-Indole), 7.85 (s, 1H, Ar-H), 7.55 (dd, *J* = 7.26, 2.43 Hz, 1H, H-Indole), 7.37 (dd, *J* = 7.41, 2.81 Hz, 1H, H-Indole), 7.25 (d, *J* = 7.41 Hz, 1H, Ar-H), 7.17 (d, *J* = 7.56 Hz, 1H, Ar-H), 7.14 (s, 1H, H-Indole), 7.12 (m, *J* = 6.5-7.5 Hz, 1H, H-Indole), 7.07 (m, *J* = 6.3-7.3 Hz, 1H, H-Indole),4.27 (t, *J* = 4.5 Hz, 1H, H-Aliphatic), 4.02 (s, 2H, H-Thiazolidinone), 1.92 (d, *J* = 4.3 Hz, 2H, H-Aliphatic); ^{13}C NMR (125 MHz, DMSO-d6): *δ* 172.3, 163.7, 163.5, 155.7, 139.3, 136.3, 132.7, 131.3, 130.2, 128.6, 127.5, 124.7, 123.5, 121.5, 121.3, 119.5, 118.5, 113.5, 111.4, 50.4, 33.7, 19.3, 17.3; HREI MS :*m/z* calcd for $C_{23}H_{17}ClF_3N_5O_2S$ $[M]^+$ 519.93 Found 519.41

3-(2,5-Dinitrophenyl)-2-((5-(3,3,3-trifluoro-2-(1H-indol-3-yl)propyl)-1,3,4-oxadiazol-2-yl)imino)thiazolidin-4-one) (Compound 11)

^{1}H NMR (600MHz, DMSO-*d6*): *δ* 12.73 (s, 1H, H-Indole), 8.82 (s, 1H, Ar-H), 8.52 (d, *J* = 7.41 Hz, 1H, Ar-H), 8.25 (d, *J* = 7.56 Hz, 1H, Ar-H), 7.55 (dd, *J* = 7.26, 2.43 Hz, 1H, H-Indole), 7.37 (dd, *J* = 7.41, 2.81 Hz, 1H, H-Indole), 7.14 (s, 1H, H-Indole), 7.12 (m, *J* = 6.5-7.5 Hz, 1H, H-Indole), 7.07 (m, *J* = 6.3-7.3 Hz, 1H, H-Indole), 3.27 (t, *J* = 4.5 Hz, 1H, H-Aliphatic), 2.13 (s, 2H, H-Thiazolidinone), 1.82 (d, *J* = 4.3 Hz, 2H, H-Aliphatic); ^{13}C NMR (125 MHz, DMSO-d6): *δ* 172.3, 163.7, 163.5, 155.7, 154.5, 148.3, 136.3, 136.1, 128.6, 127.5, 125.7, 123.5, 121.3, 120.5,

119.5, 118.5, 115.6, 113.5, 111.4, 50.4, 33.7, 19.3; HREI MS: *m/z* calcd for $C_{22}H_{14}F_3N_7O_6S$ $[M]^+$ 561.45 Found 561.13

3-(3-Nitrophenyl)-2-((5-(3,3,3-trifluoro-2-(1H-indol-3-yl)propyl)-1,3,4-oxadiazol-2-yl)imino)thiazolidin-4-one) (Compound 12)

^{1}H NMR (600MHz, DMSO-*d6*): δ 12.28 (s, 1H, H-Indole), 8.46 (s, 1H, Ar-H), 7.87 (dd, *J* = 7.41, 2.34 Hz, 1H, Ar-H), 7.74 (dd, *J* = 7.56, 2.37 Hz, 1H, Ar-H), 7.62 (dd, *J* = 7.13, 2.42 Hz, 1H, Ar-H), 7.47 (dd, *J* = 7.26, 2.43 Hz, 1H, H-Indole), 7.08 (dd, *J* = 7.41, 2.81 Hz, 1H, H-Indole), 6.85 (s, 1H, H-Indole), 6.72 (m, *J* = 6.5-7.5 Hz, 1H, H-Indole), 7.72 (m, *J* = 6.3-7.3 Hz, 1H, H-Indole), 3.53 (t, *J* = 4.5 Hz, 1H, H-Aliphatic), 2.34 (s, 2H, H-Thiazolidinone), 2.08 (d, *J* = 4.3 Hz, 2H, H-Aliphatic); ^{13}C NMR (125 MHz, DMSO-d6): δ 172.2, 167.6, 165.7, 157.5, 151.8, 148.3, 146.8, 136.8, 129.6, 126.5, 124.7, 121.7, 121.5, 112.3, 108.2, 106.6, 104.1, 51.2, 34.3, 21.7; HREI MS: *m/z* calcd for $C_{22}H_{15}F_3N_6O_4S$ $[M]^+$ 516.46 Found 516.13

3-(3-Methoxyphenyl)-2-((5-(3,3,3-trifluoro-2-(1H-indol-3-yl)propyl)-1,3,4-oxadiazol-2-yl)imino)thiazolidin-4-one) (Compound 13)

^{1}H NMR (600MHz, DMSO-*d6*): δ 12.73 (s, 1H, H-Indole), 7.55 (dd, *J* = 7.26, 2.43 Hz, 1H, H-Indole), 7.36 (dd, *J* = 7.41, 2.34 Hz, 1H, Ar-H), 7.32 (dd, *J* = 7.41, 2.81 Hz, 1H, H-Indole), 7.17 (s, 1H, H-Indole), 7.16 (dd, *J* = 7.13, 2.42 Hz, 1H, Ar-H), 7.12 (m, *J* = 6.5-7.5 Hz, 1H, H-Indole), 7.07 (m, *J* = 6.3-7.3 Hz, 1H, H-Indole), 7.05 (s, 1H, Ar-H), 7.01 (dd, *J* = 7.56, 2.37 Hz, 1H, Ar-H), 3.27 (t, *J* = 4.5 Hz, 1H, H-Aliphatic), 2.99 (s, 2H, H-Thiazolidinone), 1.74 (s, 3H, H-Aliphatic), 2.25 (d, *J* = 4.3 Hz, 2H, H-Aliphatic); ^{13}C NMR (125 MHz, DMSO-d6): δ 172.3, 163.7, 163.5, 160.5, 155.7, 136.3, 136.1, 129.3, 128.6, 127.5, 123.5, 121.3, 120.1, 119.5, 118.5, 116.7, 113.5, 111.4, 104.4, 55.5, 50.4, 33.7, 19.3; HREI MS: *m/z* calcd for $C_{23}H_{18}F_3N_5O_3S$ $[M]^+$ 501.48 Found 501.13

3-(3-Phenoxyphenyl)-2-((5-(3,3,3-trifluoro-2-(1H-indol-3-yl)propyl)-1,3,4-oxadiazol-2-yl)imino)thiazolidin-4-one) (Compound 14)

^{1}H NMR (600MHz, DMSO-*d6*): δ 10.73 (s, 1H, H-Indole), 7.55 (dd, *J* = 7.26, 2.43 Hz, 1H, H-Indole), 7.45 (dd, *J* = 7.26, 2.43 Hz, 1H, Ar-H), 7.41 (dd, *J* = 7.64, 2.32 Hz, 2H, Ar-H), 7.37 (dd, *J* = 7.41, 2.81 Hz, 1H, H-Indole), 7.19 (m, *J* = 6.5-7.5 Hz, 1H, Ar-H), 7.17 (dd, *J* = 7.66, 2.73 Hz, 1H, Ar-H), 7.14 (s, 1H, H-Indole), 7.10 (m, *J* = 6.5-7.5 Hz, 1H, H-Indole), 7.09 (s, 1H, Ar-

H), 7.07 (m, $J = 6.3\text{-}7.3$ Hz, 1H, H-Indole), 7.06 (dd, $J = 7.56, 2.18$ Hz, 2H, Ar-H), 7.04 (dd, $J = 7.64, 2.32$ Hz, 1H, Ar-H), 3.40 (t, $J = 4.5$ Hz, 1H, H-Aliphatic), 2.96 (s, 2H, H-Thiazolidinone), 2.35 (d, $J = 4.3$ Hz, 2H, H-Aliphatic); ^{13}C NMR (125 MHz, DMSO-d$_6$): δ 172.3, 163.7, 163.5, 157.5, 157.3, 155.7, 136.2, 135.5, 128.7, 128.5, 128.3, 128.1, 127.5, 123.5, 121.7, 121.5, 121.3, 119.5, 118.5, 118.3, 118.1, 114.5, 113.5, 111.4, 107.5, 50.4, 33.7, 19.3; HREI MS :*m/z* calcd for $C_{28}H_{20}F_3N_5O_3S$ $[M]^+$ 563.56 Found 563.11

3-(2,5-Difluorophenyl)-2-((5-(3,3,3-trifluoro-2-(1H-indol-3-yl)propyl)-1,3,4-oxadiazol-2-yl)imino)thiazolidin-4-one) (*Compound 15)*

^{1}H NMR (600MHz, DMSO-*d$_6$*): δ 12.19 (s, 1H, H-Indole), 8.83 (s, 1H, Ar-H), 8.57 (s, 1H, H-Indole), 8.07 (dd, $J = 7.26, 2.43$ Hz, 1H, H-Indole), 7.85 (dd, $J = 7.41, 2.81$ Hz, 1H, H-Indole), 7.72 (d, $J = 7.41,$ Hz, 1H, Ar-H), 7.49 (d, $J = 7.13$ Hz, 1H, Ar-H), 7.27 (m, $J = 6.5\text{-}7.5$ Hz, 1H, H-Indole), 7.22 (m, $J = 6.3\text{-}7.3$ Hz, 1H, H-Indole), 3.70 (t, $J = 4.5$ Hz, 1H, H-Aliphatic), 2.28 (s, 2H, H-Thiazolidinone), 1.54 (d, $J = 4.3$ Hz, 2H, H-Aliphatic); ^{13}C NMR (125 MHz, DMSO-d$_6$): δ 172.7, 161.9, 159.1, 158.1, 149.6, 141.5, 138.9, 137.9, 133.0, 132.9, 131.6, 131.0, 130.4, 129.2, 128.7, 122.7, 121.1, 54.1, 34.6, 20.6; HREI MS: *m/z* calcd for $C_{22}H_{14}F_5N_5O_2S$ $[M]^+$ 507.44 Found 507.13

S.2 Alpha-Glucosidase Activity Assay:

The inhibitory potential of Alpha-glucosidase was assessed using a well-established method [39]. Briefly, a 96-well plate was utilized, and each well received 35 µL of phosphate buffer, 31 µL of the tested compound solution (concentration range: 50-250 µg/mL), and 18 µL of [4-nitrophenyl-α-D glucopyranoside(p-NPG)] substrate. This mixture was then incubated at 37 ºC for 5 minutes. Subsequently, 16 µL of α-glucosidase (0.15 U/mL) dissolved in sodium phosphate was introduced into each well, resulting in a total volume of 100 µL. The reaction was initiated by adding 100 µL of sodium carbonate (200 mM). The absorbance at 405 nm was measured using a microplate reader. This experimental procedure was conducted in triplicate, and a control group without the tested compound was included for comparison. Acarbose served as the standard reference drug. The percentage inhibition was calculated using the following equation, where "Abs" represents absorbance."

$$\%\ \boldsymbol{inhibition} = \frac{(\textbf{Abs control} - \textbf{Abs sample})}{\textbf{Abs control}} \times \mathbf{100}$$

S.3 Alpha-Amylase Activity Assay:

The inhibitory potential of α-Amylase enzyme was evaluated through the standard methodology [40]. A test tube containing (250 μL) of the compound being tested at various concentrations (50 - 250 μg/mL), (250 μL) of [1% (w/v)] starch solution, and (250 μL) of (1U/mL) alpha-amylase solution was prepared. Post-incubation at 20°C for 3 minutes, the enzymatic process was halted by adding (500 μL) of dinitro salicylic acid (color Reagent). Following this, (250 μL) of α-amylase was immediately introduced after heating the mixture in hot water. The mixture was subsequently subjected to heating at 85°C for duration of 15 minutes, followed by a 5-minute incubation period at room temperature. (4500 μL) of distilled water was added, resulting in a final volume of (6000 μL). Spectrophotometric analysis at 540 nm was conducted to determine absorbance. A control sample devoid of the test substance was prepared, and Acarbose served as the reference drug. The % inhibition equation is used for calculation.

S.4 Docking protocol

Protein data bank (PDB) was used a medium for retrieval of crystalline structure, optimizing the structure by the removal water molecules, co-factors and hetero-atoms and computing hydrogen bonds, charges and the missing atoms. Benzothiazole based sulfonamide derivatives used for docking studies were prepared and then optimized by the use of built and Ligand Preparation module implemented in Discovery Studio 2018 (Dassault Systemes BIOVIA, USA). Gold docking tool was used for docking analysis, Ligand preparation involves generating varied tautomer's, bond order assigning and stereochemistry. Furthermore, amylase active site was surrounded by the receptor grid choosing centroid of complexed ligand (Montbretin A). Radius of 12 Å around the Montbretin A binding site was defined for enzyme active site. Accomplishment of docking calculations was achieved using Chem PLP scoring function [41].

References

39. Govindappa, M.; Hemashekhar, B.; Arthikala, M.; Rai, V.; Ramachandra, Y. L. antiinflammatory and antityrosinase activity of green synthesized silver nanoparticles using Calophyllum tomentosum leaves extract. Results in Physics 2018, 9, 400–408.
40. Ali, H.; Houghton, P. J.; Soumyanath, A. α-Amylase inhibitory activity of some Malaysian plants used to treat diabetes; with particular reference to Phyllanthus amarus. Journal of Ethnopharmacology 2006, 107, 449–455 doi: 10.1016/j.jep.2006.04.004

41. Khan, S.; Hussain, R.; Khan, Y.; Khan, T.; Ullah, F.; Felemban, S.; Khowdiary, M. M. Facile benzothiazole-triazole based thiazole derivatives as novel thymidine phosphorylase and α-glucosidase inhibitors: Experimental and computational approaches. Enzyme and Microbial Technology 2024, 110470.

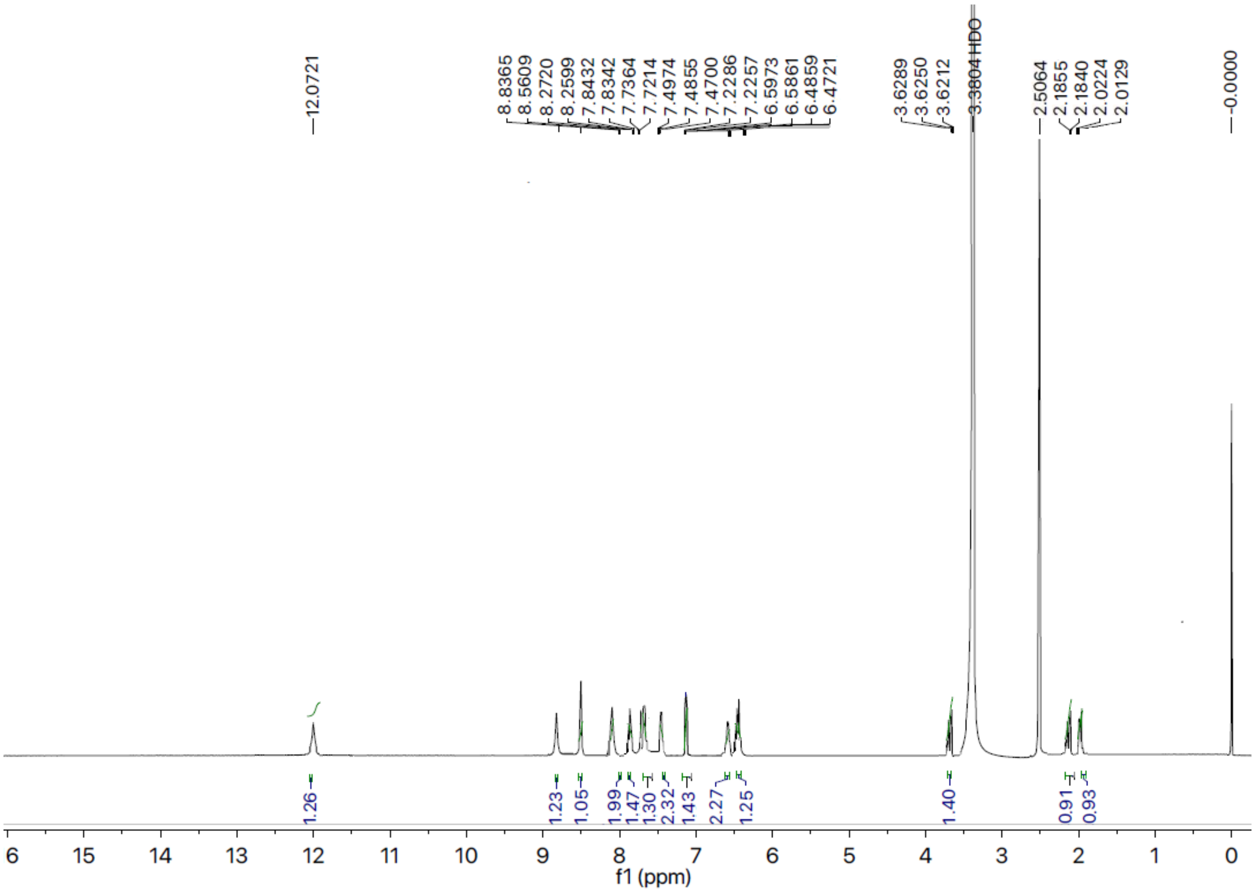

Figure S1. ^{1}HNMR for the compound **1** ***3-(4-chlorophenyl)-2-((5-(3,3,3-trifluoro-2-(1H-indol-3-yl)propyl)-1,3,4-oxadiazol-2-yl)imino)thiazolidin-4-one)***

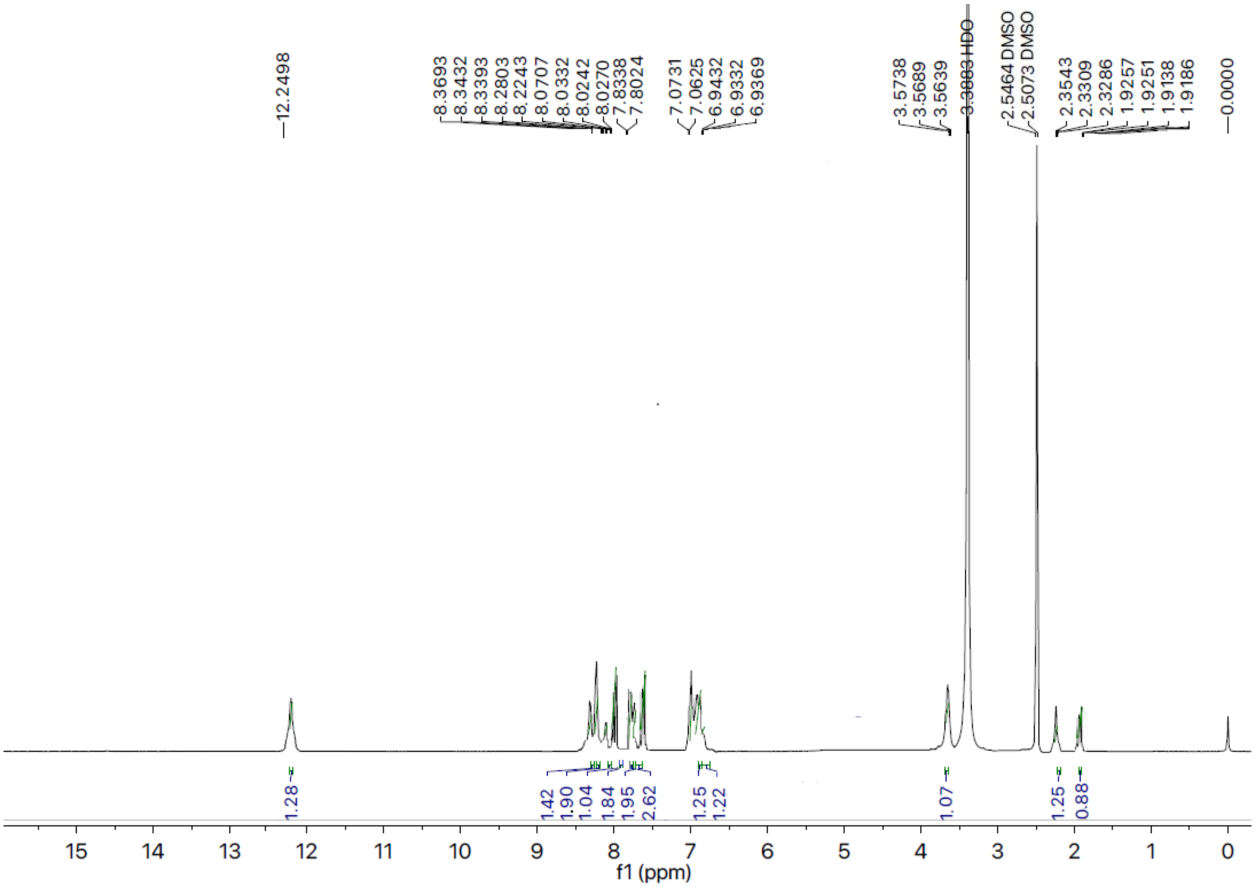

Figure S2. ^{1}HNMR for the compound **2** ***3-(4-nitrophenyl)-2-((5-(3,3,3-trifluoro-2-(1H-indol-3-yl)propyl)-1,3,4-oxadiazol-2-yl)imino)thiazolidin-4-one)***

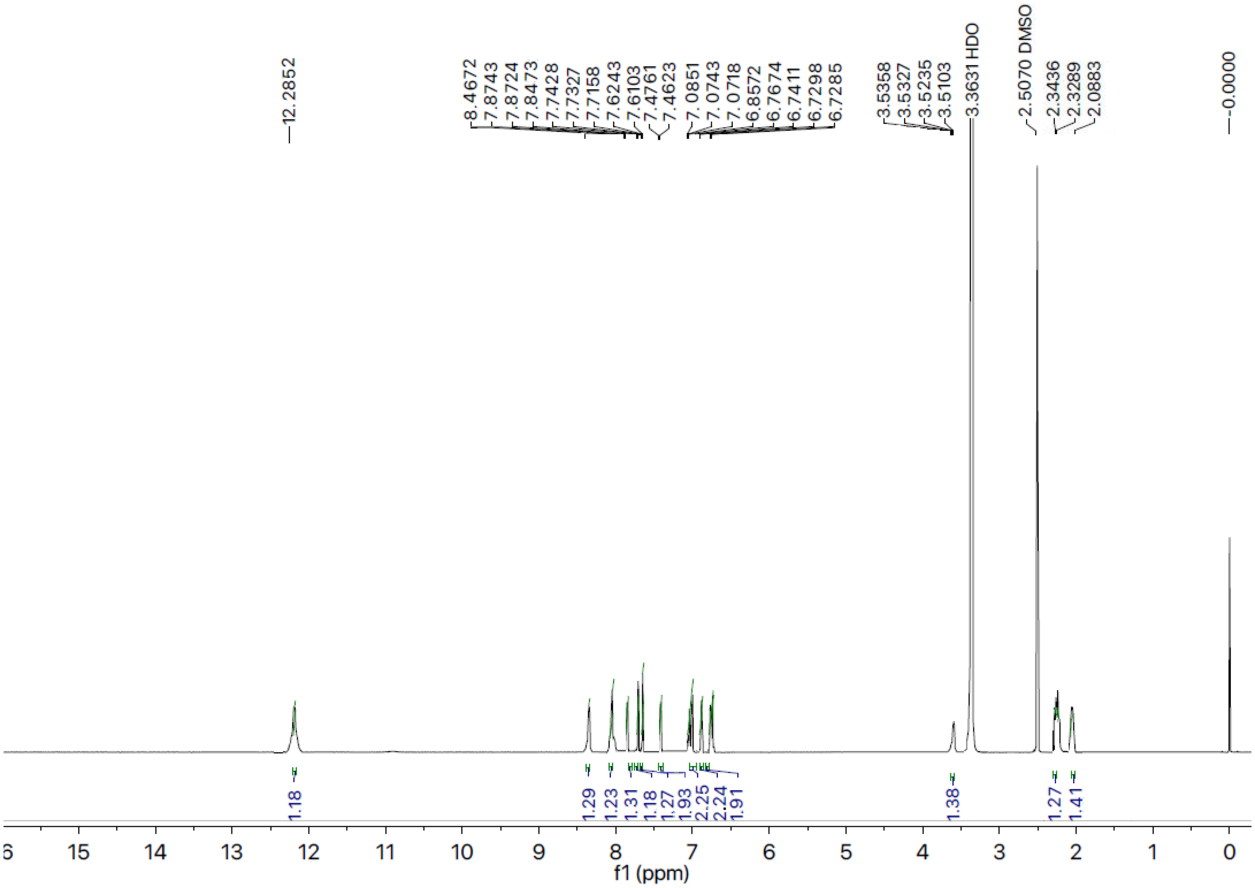

Figure S3. ^{1}HNMR for the compound **12** ***3-(3-nitrophenyl)-2-((5-(3,3,3-trifluoro-2-(1H-indol-3-yl)propyl)-1,3,4-oxadiazol-2-yl)imino)thiazolidin-4-one)***

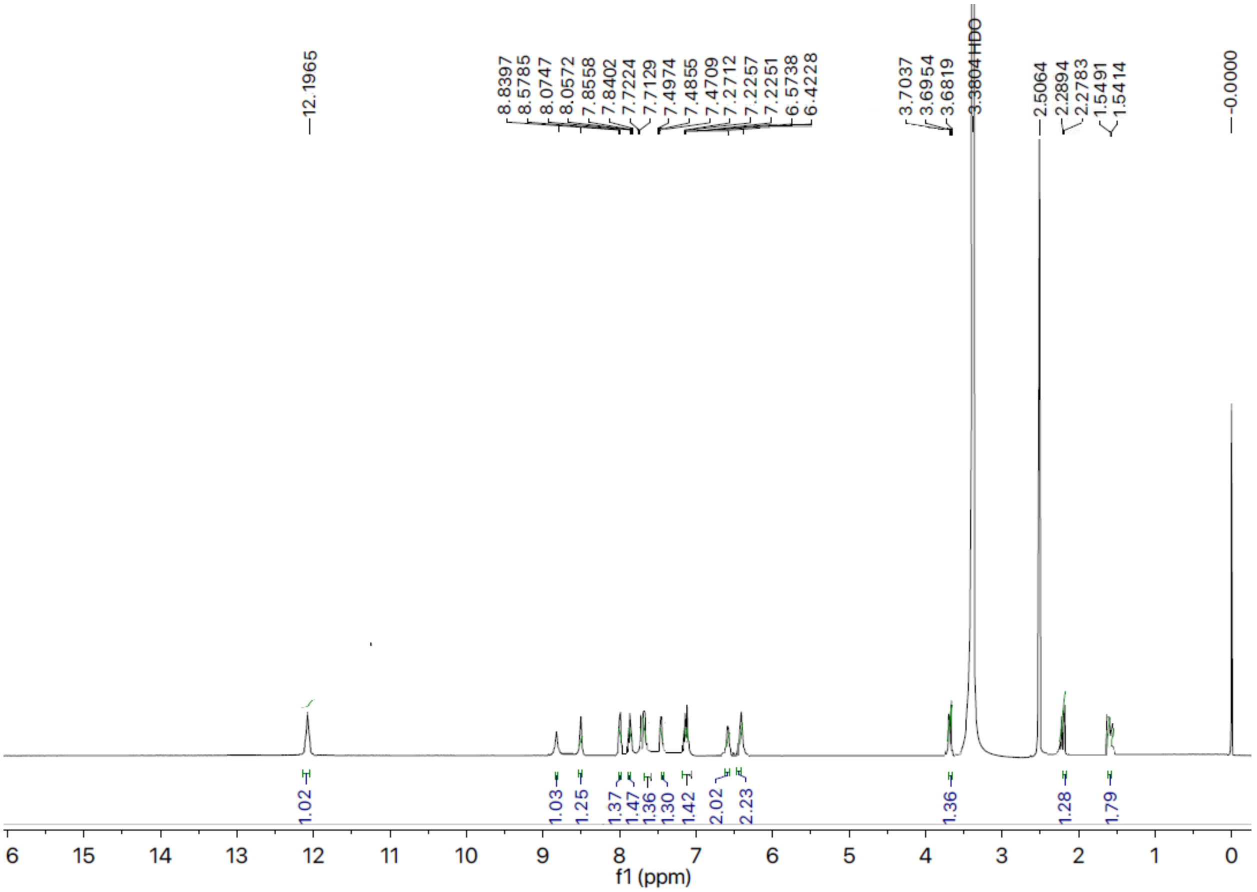

Figure S4. ^{1}HNMR for the compound **15** ***3-(2,5-difluorophenyl)-2-((5-(3,3,3-trifluoro-2-(1H-indol-3-yl)propyl)-1,3,4-oxadiazol-2-yl)imino)thiazolidin-4-one)***

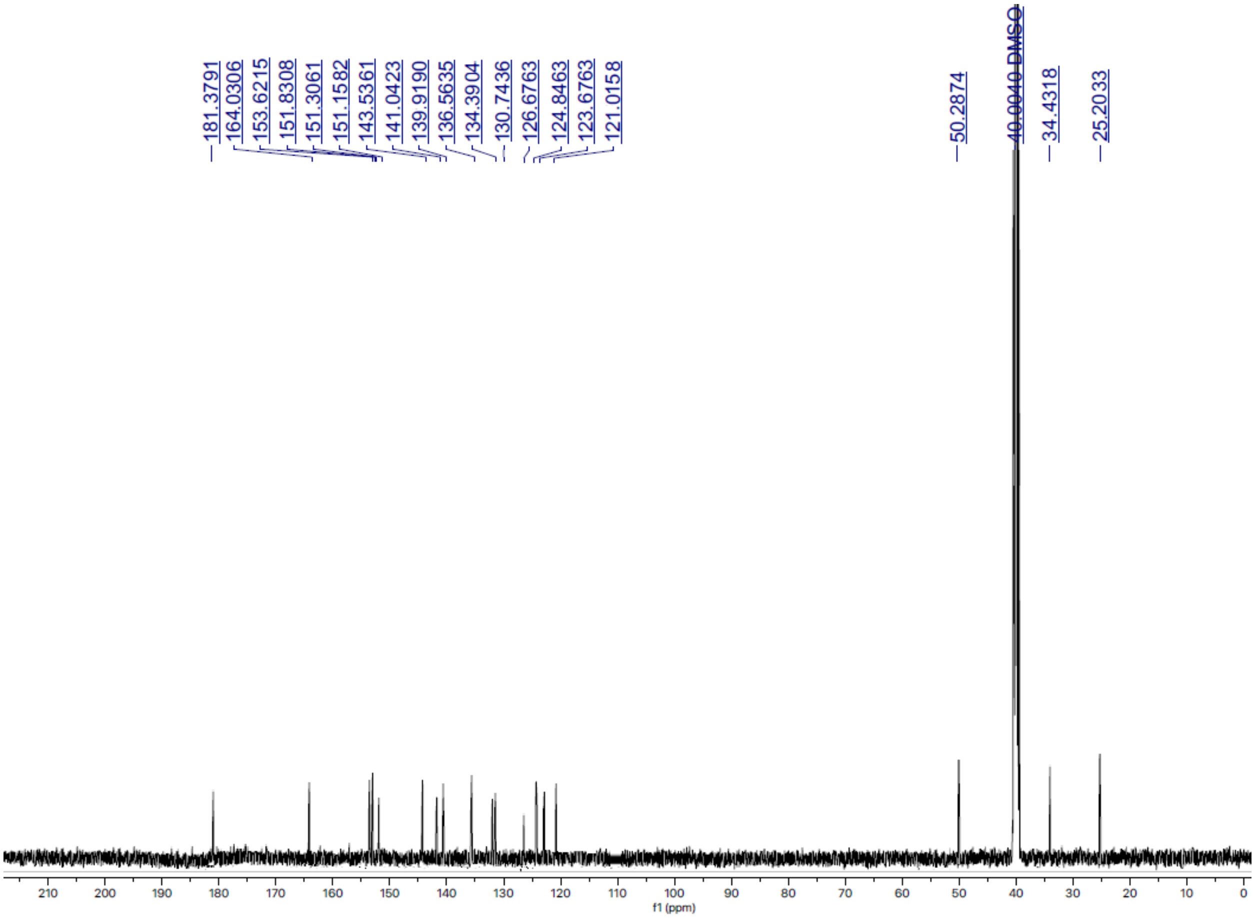

Figure S5. ^{13}CNMR for the compound **1** ***3-(4-chlorophenyl)-2-((5-(3,3,3-trifluoro-2-(1H-indol-3-yl)propyl)-1,3,4-oxadiazol-2-yl)imino)thiazolidin-4-one)***

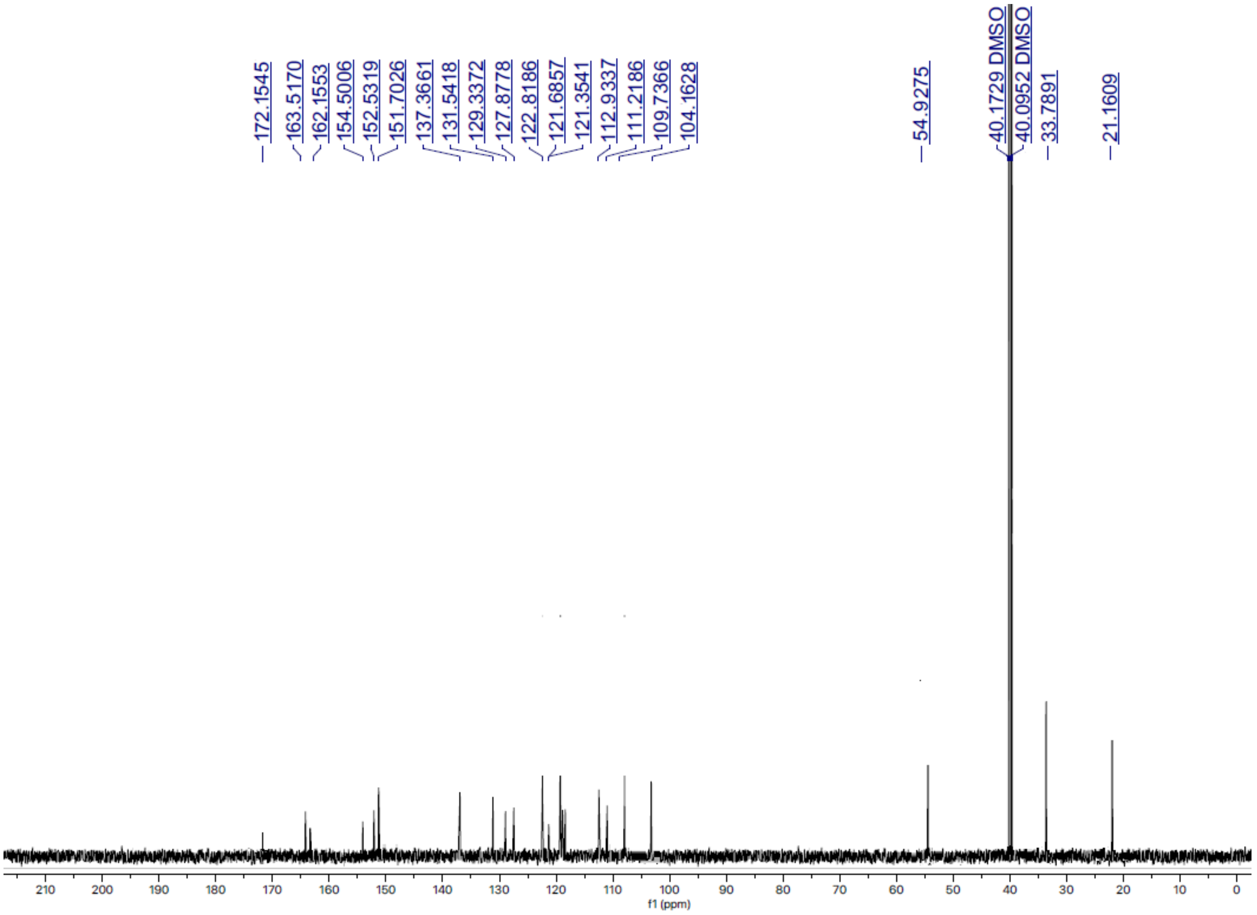

Figure S6. ^{13}CNMR for the compound **6** ***3-(4-nitrophenyl)-2-((5-(3,3,3-trifluoro-2-(1H-indol-3-yl)propyl)-1,3,4-oxadiazol-2-yl)imino)thiazolidin-4-one)***

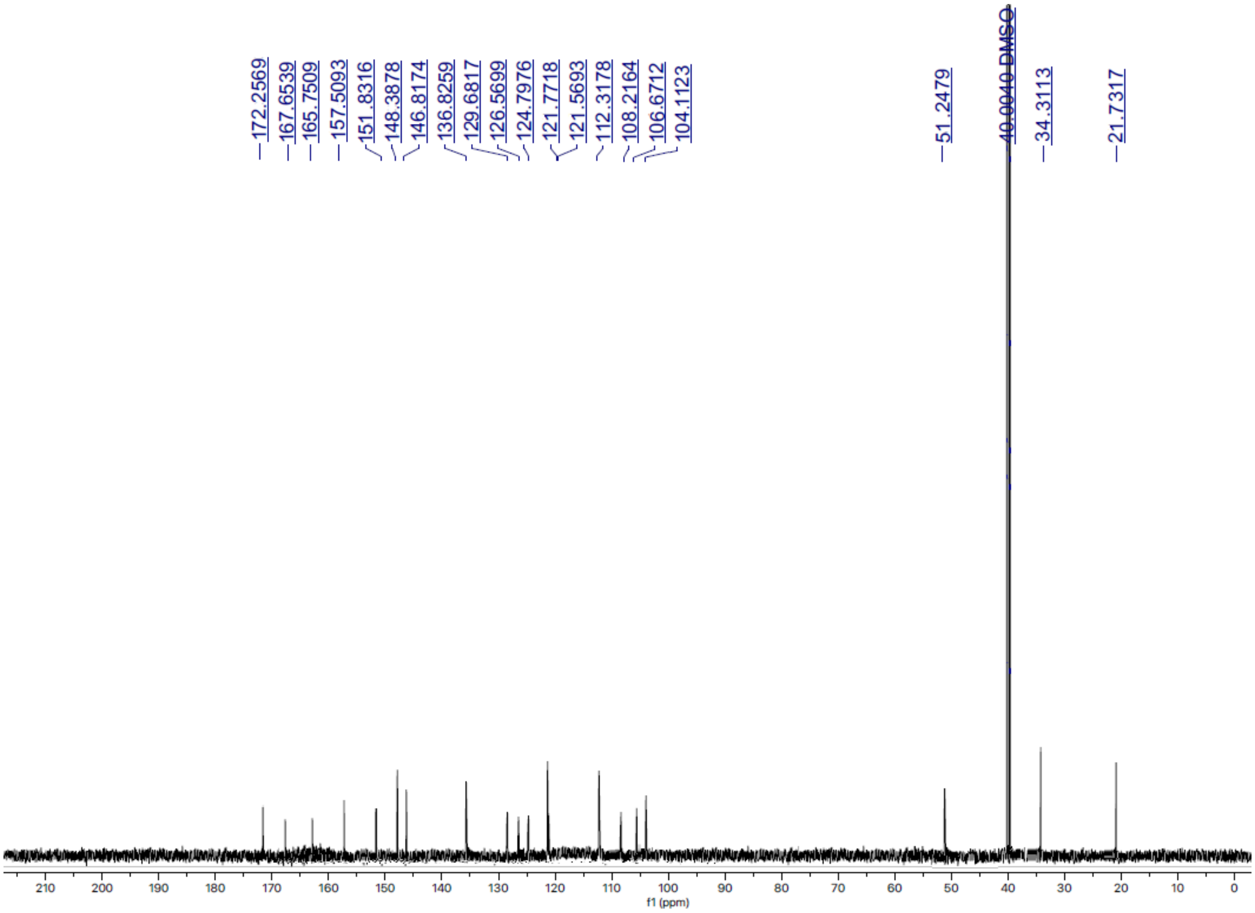

Figure S7. ^{13}CNMR for the compound **12** ***3-(3-nitrophenyl)-2-((5-(3,3,3-trifluoro-2-(1H-indol-3-yl)propyl)-1,3,4-oxadiazol-2-yl)imino)thiazolidin-4-one)***

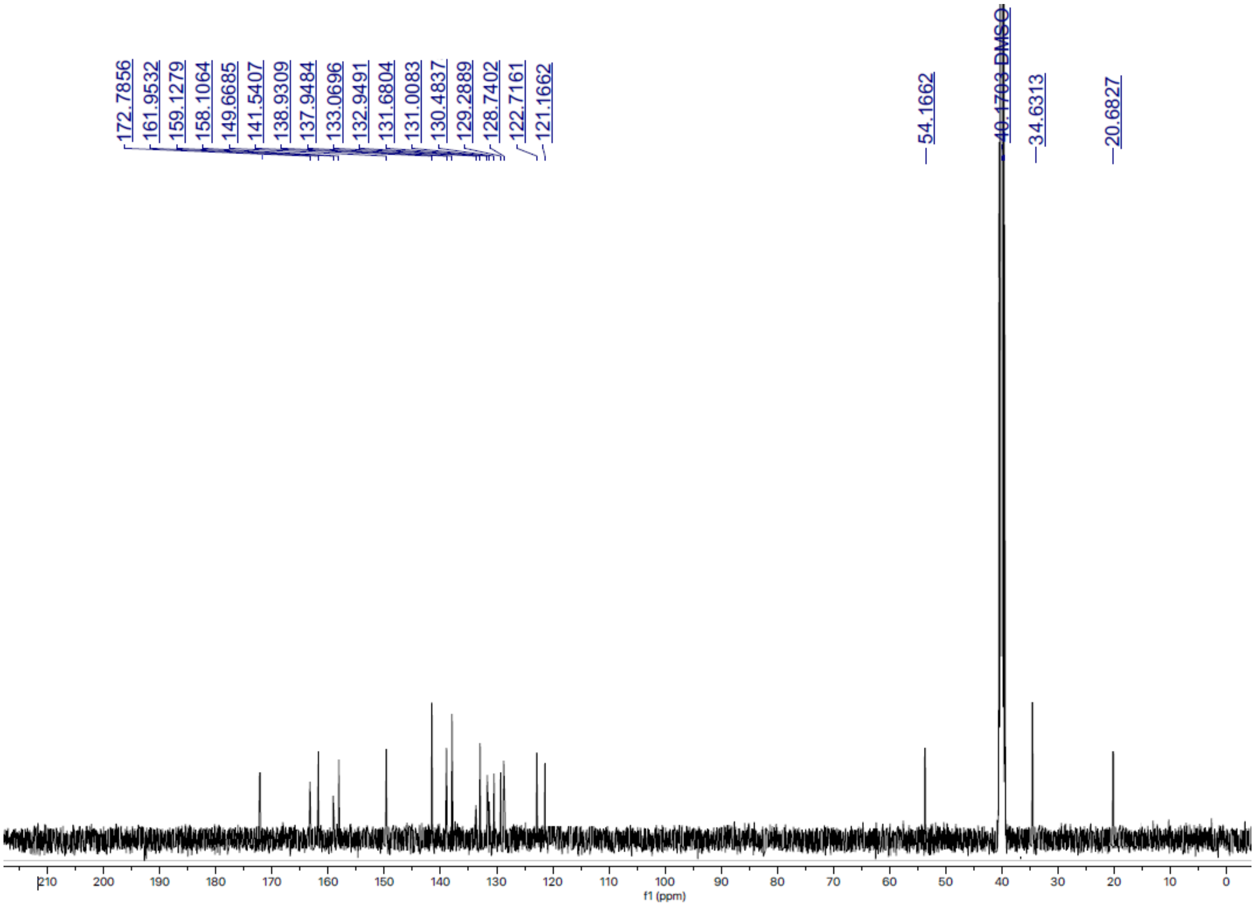

Figure S8. ^{13}CNMR for the compound **15** ***3-(2,5-difluorophenyl)-2-((5-(3,3,3-trifluoro-2-(1H-indol-3-yl)propyl)-1,3,4-oxadiazol-2-yl)imino)thiazolidin-4-one)***

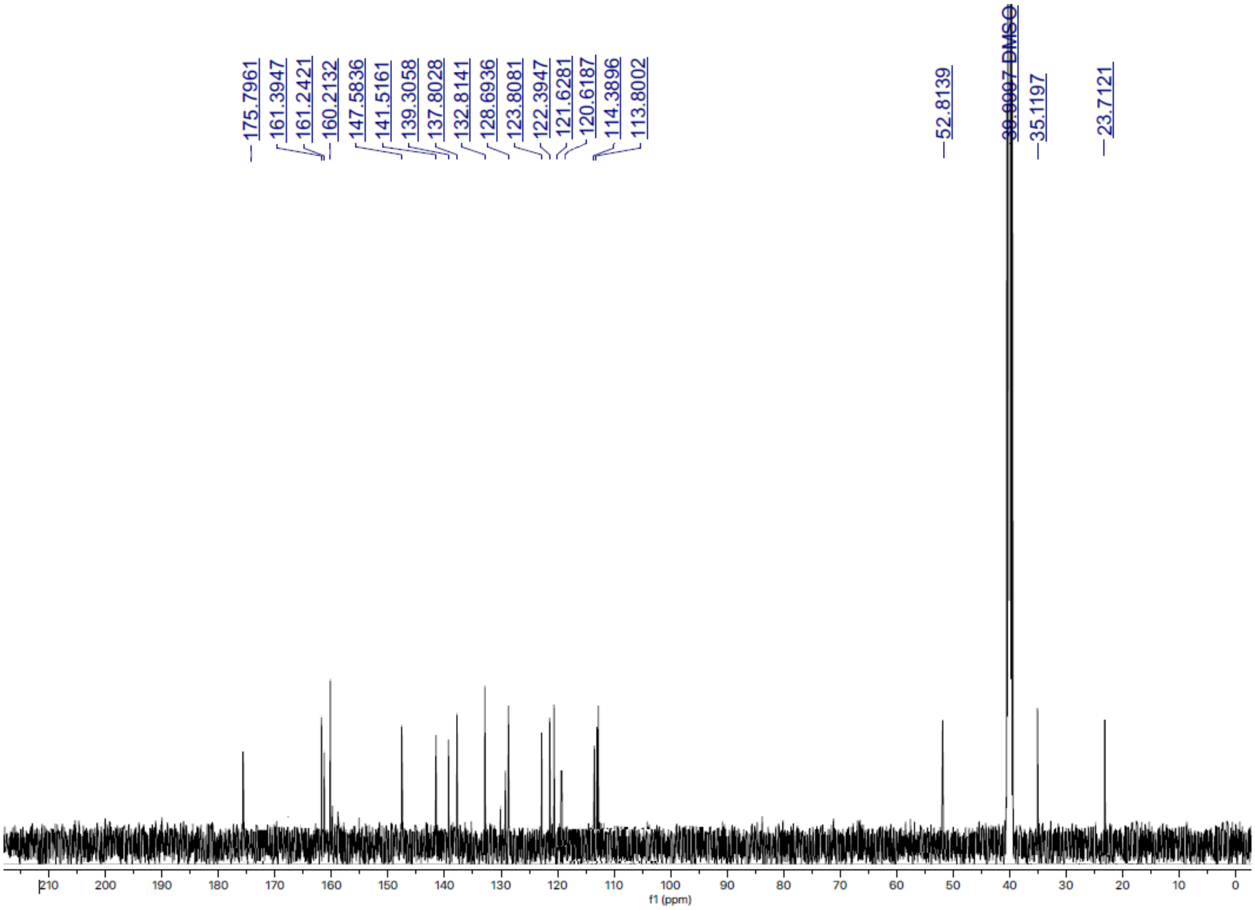

Figure S9. ^{13}CNMR for the compound **7** ***3-(4-fluorophenyl)-2-((5-(3,3,3-trifluoro-2-(1H-indol-3-yl)propyl)-1,3,4-oxadiazol-2-yl)imino)thiazolidin-4-one)***

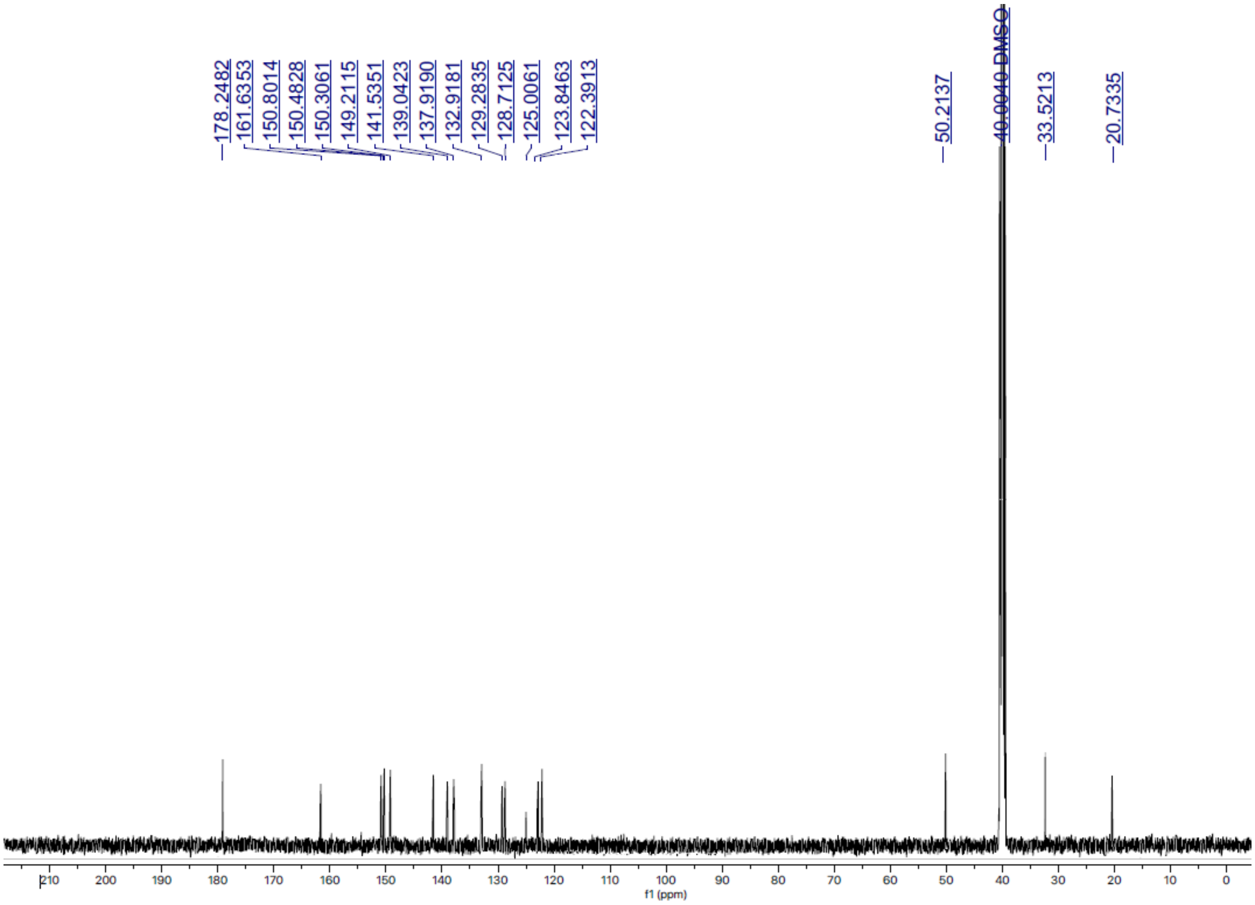

Figure S10. ^{13}CNMR for the compound **5** ***3-(4-bromophenyl)-2-((5-(3,3,3-trifluoro-2-(1H-indol-3-yl)propyl)-1,3,4-oxadiazol-2-yl)imino)thiazolidin-4-one)***
